# Systematic Review and Meta-Analysis of Insulin Dose and Route of Administration Regimens for Diabetic Ketoacidosis in Children and Adolescents

**DOI:** 10.3390/jcm14217792

**Published:** 2025-11-03

**Authors:** Hiba Idrees, Fozia Memon, Ridwa Alam, Muhammad Talal, Aqsa Ishaq, Fatima Amjad, Eddy Lang, Sajid B. Soofi, Shabina Ariff

**Affiliations:** 1 Centre of Excellence in Women and Child Health, Aga Khan University, Karachi 74800, Pakistanridwa.alam@aku.edu (R.A.); muhammadtalal1999@gmail.com (M.T.);; 2 Department of Paediatrics and Child Health, Aga Khan University, Karachi 74800, Pakistan; 3 Cumming School of Medicine, University of Calgary, Calgary, AB T2N 2T8, Canada

**Keywords:** diabetic ketoacidosis, insulin regimens, child health, adolescent health

## Abstract

**Background:** Non-communicable diseases represent a major global health challenge. Among these, diabetic ketoacidosis (DKA), an acute complication of type 1 diabetes mellitus in children and adolescents, significantly contributes to worldwide morbidity and mortality. Effective management of DKA relies on adequate insulin therapy, but variability in dosing, administration, and frequency leads to increased risk of complications and delayed DKA resolution. We conducted a systematic review of randomized controlled trials (RCTs) to evaluate the insulin dose and route of administration regimens for managing pediatric DKA. **Methods:** This review followed the PRISMA guidelines and was registered on PROSPERO (CRD42024568747). A comprehensive search of PubMed, CINAHL, Cochrane Library, and Scopus identified studies examining insulin regimens in pediatric DKA. Eligible studies were assessed for risk of bias using the Cochrane’s Risk of Bias (RoB-2) tool, and data were pooled using Review Manager for meta-analysis. Outcomes included morbidity (cerebral injury, hypoglycemia, hypokalemia), mortality, hospital stay, and adverse events. The quality of evidence was evaluated using the Grading of Recommendations Assessment, Development and Evaluation (GRADE) criteria. This review was commissioned by the WHO for the development of consolidated guidelines on common childhood illnesses. **Results:** Twelve RCTs, involving 530 participants, were included. A lower insulin dose (0.05 U/kg/h) was associated with a significantly reduced risk of hypoglycemia (RR = 0.39, 95% CI: 0.18–0.88, *p* = 0.02) and hypokalemia (RR = 0.54, 95% CI: 0.33,0.89, *p* = 0.01) compared to 0.1 U/kg/h. There were no significant differences in mortality and length of hospital stay between the dosing regimens. Additionally, no significant differences were observed in the incidence of cerebral injury and other adverse events. **Conclusions:** Findings suggest that lower insulin doses may reduce the risks of hypoglycemia and hypokalemia in children with mild-to-moderate DKA without increasing the risk of mortality, cerebral injury, or length of hospital stay. Further studies are needed to provide an evidence-based core outcome set and refine insulin dosing strategies across the full spectrum of disease severity.

## 1. Introduction

Diabetic Ketoacidosis (DKA) is potentially the most life-threatening acute complication of type 1 diabetes mellitus (T1DM) in children and adolescents [1]. It is a complex metabolic derangement characterized by hyperglycemia, acidemia, and ketonemia. The underlying pathophysiology for the disease is characterized by a significant reduction in effective circulating insulin coupled with elevated counterregulatory hormones, including glucagon, catecholamines, growth hormone, and cortisol [2]. Among children with T1DM, DKA remains the leading cause of mortality, accounting for 50% of all deaths [1]. Mortality rates range from 0.15 to 0.31% in high-income countries to 3.4–13.4% in low- and middle-income countries, underscoring global disparities in care and outcomes [3].

Timely and effective diagnosis and management of DKA are crucial to prevent life-threatening complications such as cerebral injury, which carries a high mortality rate of 20–90% across studies. The primary initial treatment for DKA involves prompt fluid resuscitation, followed by insulin therapy [4,5,6]. While fluid therapy can improve perfusion and modestly lower glucose, it does not address the high-anion-gap metabolic acidosis characteristic of DKA. Insulin is therefore required not only for lowering blood glucose, but just as importantly, for suppressing ketogenesis, thereby facilitating the resolution of the acidosis [7,8,9,10].

Current guidelines strongly suggest that low-dose (0.05 unit/kilogram/hour (U/kg/h) to 0.1 U/kg/h) intravenous (IV) insulin is an effective regimen for managing DKA with minimal adverse events [11,12,13,14]. As such, this approach is considered the standard of care until the resolution of the metabolic features of DKA is obtained (ketonemia and acidosis), which often takes longer than normalization of blood glucose concentrations [15]. Consequently, insulin treatment continues during this period, and IV glucose is commonly required to prevent hypoglycemia while insulin administration is in progress, to arrest ketogenesis and reverse metabolic acidosis. However, in resource-limited settings or for patients with mild-to-moderate DKA, subcutaneous (SC) or intramuscular (IM) administration of short- or rapid-acting insulin is a practical and effective alternative [16]. As a result, variability in insulin dosing and administration among healthcare settings is influenced by factors such as available resources, DKA severity and discrepancies in treatment protocol at different centers [12,17]. Given this variability in insulin management, there is a crucial need for standardized evidence-based guidelines tailored to the age-specific management of pediatric DKA. The Integrated Management of Childhood Illness (IMCI) guidelines and the pocketbook for hospital care for children by the World Health Organization (WHO) provide comprehensive clinical recommendations for outpatient and inpatient care for sick children. However, neither provides explicit recommendations for children and adolescents with T1DM, particularly those presenting with DKA. This systematic review was therefore commissioned by the WHO to inform the development of consolidated guidelines on the management of common childhood illnesses. In this review, we evaluate the optimal dose, route, and frequency of insulin regimens for managing DKA in pediatric populations.

## 2. Methods

### 2.1. Protocol Registration and Guidelines

The systematic review adhered to the 2020 Preferred Reporting Items for Systematic Reviews and Meta-Analyses (PRISMA) guidelines to identify, appraise, and synthesize relevant studies. The protocol for this review was registered with the International Prospective Register of Systematic Reviews (PROSPERO: CRD42024568747).

### 2.2. Eligibility Criteria

Studies were included if they involved children and adolescents aged 1 to 19 years with DKA, examined insulin regimens as an intervention, and reported pre-specified outcomes. Randomized controlled trials (RCTs) were included. Studies were excluded if they lacked a clear focus on insulin treatment or involved participants with non-DKA conditions. Detailed eligibility criteria are mentioned in Table 1.

### 2.3. Outcomes

The outcomes assessed in this review included the following:

1. **Morbidity**:
  - **Cerebral injury** was assessed using the Glasgow Coma Scale (GCS) or deterioration in neurological status.
  - **Hypoglycemia** was defined according to varying glucose thresholds.
  - **Hypokalemia** was defined according to varying serum potassium levels.
2. **Overall morbidity** was defined as a composite of cerebral injury, hypoglycemia, and hypokalemia.
3. **All-cause mortality** represented the total number of deaths during the study period.
4. **Length of hospital stay** measured in hours or days.
5. **Adverse events** were defined as any complications during treatment other than hypoglycemia, hypokalemia, and cerebral injury, as reported by study authors.

### 2.4. Search Methods

Relevant studies were identified through a comprehensive search of PubMed, CINAHL, Wiley Cochrane Library, and Scopus using a predefined strategy (see Supplementary File Supplementary S1). The search strategy was developed using free-text and MeSH terms to retrieve eligible studies. No date restrictions were applied, and only studies in English were included. Clinical trial registries, including clinicaltrials.gov and the WHO International Clinical Trials Registry Platform (ICTRP), as well as bibliographies of previous relevant systematic reviews and all included studies, were searched to identify any relevant studies.

### 2.5. Data Collection and Analysis

#### 2.5.1. Selection of Studies

All records identified by the search were imported into Covidence for screening (Covidence systematic review software (www.covidence.org), version Veritas Health Innovation, Melbourne, Australia). After removing duplicate entries, two reviewers independently assessed the titles and abstracts of the studies. Studies meeting eligibility criteria proceeded to full-text screening, with disagreements resolved by discussion or consultation with a third reviewer. Reasons for exclusion at this stage were documented.

#### 2.5.2. Data Extraction and Management

Data were extracted independently by two reviewers into a pre-formed extraction sheet developed on Covidence. The extracted data was checked for accuracy, and any discrepancies were resolved by discussion and consulting with a third reviewer. Missing or unpublished data were requested from study authors, and articles that could not be retrieved after exhaustive attempts were marked as not retrieved. A similar process was conducted for studies identified through the registries.

### 2.6. Quality Assessment

The quality of the included RCTs was assessed using the Cochrane Risk of Bias tool II (RoB-2) [18]. Two reviewers independently assessed the studies and evaluated the risk of bias for each outcome, with disagreements resolved through discussion or consultation. Risk was categorized as low, high, or some concerns across five domains: randomization process, deviations from intended interventions, missing outcome data, measurement of outcomes, and selection of the reported result.

### 2.7. Statistical Analysis

Meta-analyses were performed using Review Manager (RevMan) 5.4.1 to quantitatively pool studies where appropriate. Mean differences (MDs) with 95% confidence intervals (CIs) were used for continuous outcomes, and risk ratios (RRs) with 95% CIs were used for dichotomous outcomes. Hozo’s method was employed to estimate means and standard deviations when only medians and ranges/interquartile ranges were reported [19]. A random-effects model was applied, and forest plots were created for all outcomes. The results of trials that provided data unsuitable for inclusion in pooled analyses were summarized narratively.

### 2.8. Assessment of Heterogeneity

Statistical heterogeneity was assessed by I^2^ and by visual inspection of forest plots to detect non-overlapping CIs. Based on prior clinical knowledge, clinical and methodological heterogeneity in included studies was expected. Had heterogeneity been found, an attempt would have been made to determine possible reasons for it through subgroup analysis. This was not possible due to insufficient data.

### 2.9. Certainty of Evidence

The certainty of the body of evidence for each outcome was assessed using the Grading of Recommendations Assessment, Development and Evaluation (GRADE) approach. Summary of findings tables were generated to present the quality of evidence for each outcome, evaluated based on GRADE criteria [20], including risk of bias, directness of evidence, heterogeneity, precision of effect estimates, and publication bias.

Certainty was downgraded by one level for studies with overall “some concerns” of bias and by two levels for studies with a high risk of bias. Heterogeneity was considered based on the magnitude and direction of inconsistency, and certainty was downgraded accordingly. The line of no effect was defined as a minimal clinically important difference for all outcomes. For imprecision, evidence was downgraded by one level if the optimal information size (OIS), defined as 300 participants, was not met or the 95% CI crossed the line of no effect, and downgraded twice if both conditions were met. Indirectness was not a factor for downgrading, as all included studies directly addressed the research question and were conducted in tertiary care settings. Based on these assessments, the quality of evidence for each outcome was rated as “high,” “moderate,” “low,” or “very low.”

## 3. Results

**Figure 1:**
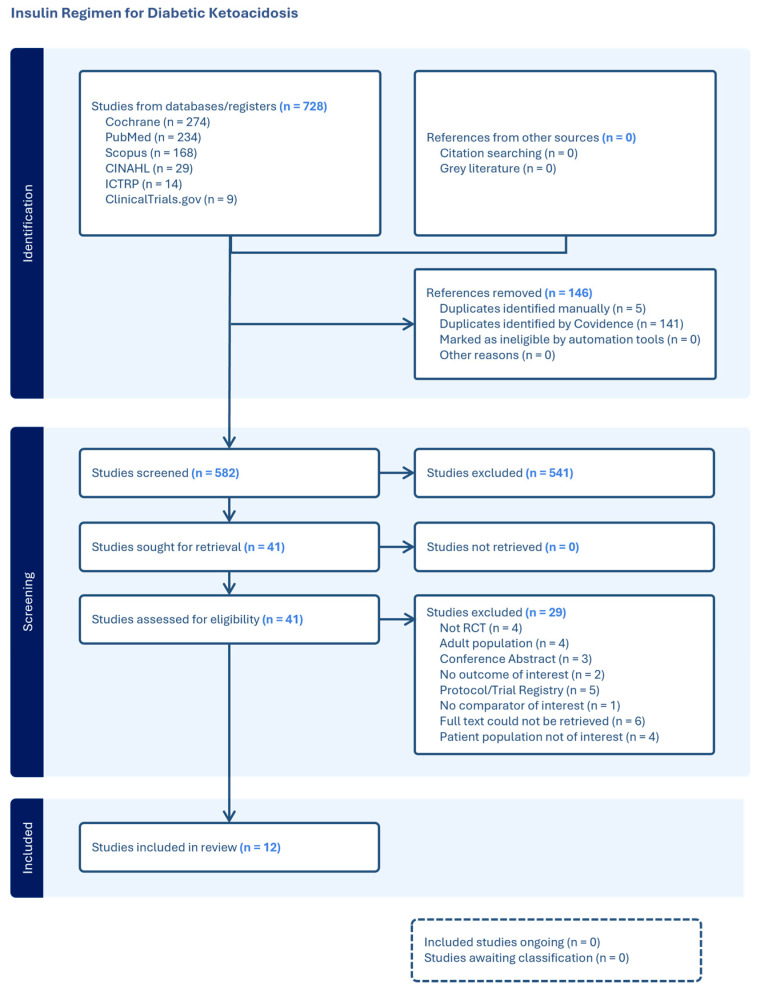
PRISMA Flow Chart.

Our search of the literature, performed on 12 June 2024 across electronic databases, identified 728 studies. After removing duplicates, 582 studies remained. Of these, 541 were excluded as irrelevant, leaving 41 studies for full-text screening. As outlined in the PRISMA flow chart (Figure 1), the most frequent reason for exclusion during full-text screening was the lack of retrievable full texts.

### 3.1. Study Characteristics

A total of 12 RCTs [21,22,23,24,25,26,27,28,29,30,31,32], comprising 530 participants aged 0 to 19 years, met the inclusion criteria for this systematic review. Study sizes ranged from 10 to 108 participants, and the trials were conducted across various countries, notably the USA, India, Iran, and Brazil. All but one study was open-label [29]. Detailed study characteristics are provided in Table 2.

### 3.2. Intervention and Therapeutic Approaches

The diagnostic criteria for DKA varied across studies but generally included hyperglycemia (blood glucose > 200 mg/dL to >400 mg/dL, acidosis (pH < 7.3), low bicarbonate levels, and often ketonemia or ketonuria. The intervention group typically received lower insulin doses ranging from 0.05 to 0.25 U/kg/h. The most common dose used was 0.1 U/kg/h of intravenous IV regular insulin. Some studies also used SC [22,23,24,28,30,31] or IM insulin [27], including Aspart, Lispro, Glargine and Detemir. In cases where basal (long-acting) insulins were used, they were administered as part of study protocols evaluating the addition of basal insulin during the transition phase from IV to SC therapy, rather than for acute DKA management. In contrast, control groups received doses ranging from 0.05 to 2.2 U/kg/h. The most common comparison involved 0.1 U/kg/h IV. Studies also used regular, crystalline, or porcine insulin via IV, SC, or combined routes.

Fluid therapy was largely consistent across studies, involving isotonic saline [21,22,23,31], often combined with 0.45% saline [25,26,27,29,30,32] for maintenance, or adjusted based on urine output. Potassium infusion with potassium chloride (KCl) at 40 mEq/L maintained serum potassium levels between 3.5 and 5.5 mEq/L. Glucose or dextrose was added to IV fluids when blood glucose fell below 250 mg/dL, with higher concentrations (10–12.5%) used to prevent hypoglycemia during insulin therapy, adjusting based on blood glucose levels or rate of decline. Further details of the interventions can be found in (see Supplementary File Supplementary S2).

### 3.3. Risk of Bias

For cerebral edema, five studies had some concerns [22,27,30,31,32], and three were high risk [24,25,26] due to randomization and outcome measurement issues. For hypoglycemia, two studies had low risk [26,29], seven had some concerns [21,22,24,27,28,31,32], and one had a high risk [23] due to randomization concerns. Hypokalemia followed a similar pattern to hypoglycemia, with two low-risk studies [26,29], six with some concerns [21,22,24,27,31,32], and two at high risk [23,28] related to randomization. For mortality, all studies had some concerns [21,22,26,30,32] except one with a low risk of bias [29]. Studies reporting hospital stay [30] and adverse events [22,27,30] had some concerns, with high risk linked to intervention deviations and missing data in one study [31] (see Supplementary File Supplementary S3: Figures S1–S6).

### 3.4. Comparisons Identified for Analysis

The included studies provided limited opportunities for comparisons based on insulin dose, route, and frequency. For dose, two primary comparisons were identified: the first was 0.05 U/kg/h IV insulin versus 0.1 U/kg/h IV insulin, which included three studies [26,29,32]. The second was 0.05–0.1 U/kg/h IV insulin versus > 0.1 U/kg SC insulin administered every two hours, which included two studies [22,30].

For the route of insulin administration, IV insulin was compared with SC. While no studies reported our predefined frequency for insulin delivery, we found studies comparing insulin delivery every two hours with continuous infusion instead. Hence, the insulin regimen of 0.05–0.1 U/kg/h IV insulin versus > 0.1 U/kg SC insulin administered every two hours was used for both the route and frequency comparisons.

The following seven additional studies included regimens that could not directly be compared due to differences in dose, route, or frequency:

- 0.05–0.1 U/kg/h IV + 0.5 U/kg SC versus 0.05–0.1 U/kg/h IV [31];
- 0.1 U/kg/2 h IM vs. 1.0 U/kg/4 h SC + IV [27];
- 0.1 U/kg/h IV vs. 1.0 U/kg/h IV [21];
- 0.1 U/kg/h IV vs. 1.0–2.2 U/kg SC every 3 h [24];
- 0.25 U/kg IV bolus + 0.1 U/kg/h IV vs. 2.0 U/kg IV + SC [28];
- 0.1 U/kg/h IV vs. 0.9–1.8 U/kg/h IV + SC [23];
- 0.1 U/kg/h IV with bolus dose vs. 0.1 U/kg/h IV without bolus dose [25].

Forest plots for all dose, frequency and route of administration comparisons are provided in Supplementary File: Supplementary S4.

### 3.5. Outcomes

#### 3.5.1. Morbidity: Cerebral Injury, Hypoglycemia, and Hypokalemia

Eight studies [22,24,25,26,27,29,31,32] reported cerebral injury, using varying diagnostic criteria, ranging from clinical assessments alone to neuroimaging. No statistically significant differences were observed in the incidence of cerebral injury across insulin regimens.

10 studies [21,22,24,25,26,27,28,29,31,32] reported hypoglycemia, using a variety of plasma glucose thresholds (ranging from 40 to 100 mg/dL). In the insulin dose comparison, hypoglycemia was found to be 61% less likely in the 0.05 U/kg/h group compared to the 0.1 U/kg/h group (RR = 0.39, 95% CI: 0.18 to 0.88, *p* = 0.02, I^2^ = 0%). However, we did not find any significant differences when comparing insulin routes or frequency between the two groups.

Hypokalemia was reported in 10 studies [21,22,24,25,26,27,28,29,31,32] with varying thresholds (ranging from <3 to 3.5 mmol/L). For dose, hypokalemia was 46% less likely in the 0.05 U/kg/h group compared to the 0.1 U/kg/h group (RR = 0.54, 95% CI: 0.33 to 0.89, *p* = 0.01, I^2^ = 0%). No significant differences were found in route or frequency comparisons between the two groups.

#### 3.5.2. Mortality

Six studies [21,22,26,29,30,32] addressed mortality outcomes. However, no deaths were recorded in any group.

#### 3.5.3. Hospital Stay

Two studies [30,31] examined the length of hospital stay, but only one provided comparable data by including two distinct patient populations (mild and moderate DKA). This study compared two insulin regimens: >0.1 U/kg SC insulin administered every 2 h versus 0.05–0.1 U/kg/h IV insulin. The analysis found no statistically significant difference in hospital stay between the two regimens (RR = 10.44, 95% CI: −17.79 to 38.66, *p* = 0.47, I^2^ = 92%).

#### 3.5.4. Adverse Events

Three studies [22,27,30] reported adverse events, using author-defined criteria such as hyperchloremia and hypernatremia. No significant differences were observed between insulin regimens.

### 3.6. Certainty of Evidence (GRADE)

For the comparison of 0.05 U/kg/h IV insulin versus 0.1 U/kg/h IV insulin [26,29,32], the evidence for hypokalemia was of moderate quality, while the evidence for hypoglycemia was of low certainty. Additionally, both cerebral edema and overall morbidity had very low certainty of evidence (Supplementary S5: Table S1). In the comparison of 0.05–0.1 U/kg/h IV insulin versus > 0.1 U/kg SC insulin every 2 h [22,30], the certainty of evidence for hypoglycemia and hospital stay was very low (Supplementary S5: Tables S2 and S3). Similarly, in the comparison of insulin administration frequency (every 2 h versus continuous infusion) [22,30], the evidence for hypoglycemia and hospital stay was also of very low certainty (Supplementary S5: Table S4). For studies with insulin regimens that were not directly comparable [21,23,24,25,27,28,31], the GRADE assessment was rated low to very low certainty of evidence for all outcomes reported (Supplementary S5: Tables S5–S11).

## 4. Discussion

Managing pediatric DKA requires balancing effective treatment with minimizing complications. This systematic review and meta-analysis evaluated insulin regimens for mild-to-moderate pediatric DKA, focusing on variations in dosing, administration routes, and delivery frequencies in children and adolescents. Our findings revealed that an initial intravenous insulin infusion dose of 0.05 U/kg/h is associated with significantly lower rates of hypoglycemia and hypokalemia compared to the conventional 0.1 U/kg/h dose in pediatric DKA, with no apparent differences in reported rates of cerebral injury, mortality, hospital stay, or other adverse events.

These results align with a growing body of evidence, including a recent meta-analysis [33], that supports the efficacy of lower-dose insulin (0.05 U/kg/h) in suppressing ketogenesis and lowering glucose while substantially reducing iatrogenic complications like hypoglycemia and hypokalemia. Most episodes of hypoglycemia reported across studies were mild and rapidly corrected, without sequelae or impact on hospital stay or neurological outcomes. The consistency of these findings across multiple systematic reviews strengthens the evidence that the standard 0.1 U/kg/h insulin dose may be higher than necessary for most children, and that a dose reduction can enhance safety without compromising clinical effectiveness [34]. However, we found no significant differences in the rates of cerebral injury and mortality between dosing strategies, probably due to a low incidence of these outcomes across the included studies. The low incidence of these events limits the power to definitively detect or rule out differences. Our findings support existing recommendations [14,35] that endorse continuous IV insulin infusion as first-line therapy. While the conventional starting dose is often cited as 0.1 U/kg/h after initial fluid resuscitation, ISPAD notably acknowledges a broader acceptable range of 0.05 to 0.1 U/kg/h, with the lower dose considered appropriate for less acidotic patients [35]. This aligns with our observation that lower doses reduce complications without compromising efficacy.

For route of administration, IV insulin was compared to SC insulin using the same regimens (0.05–0.1 U/kg/h IV insulin versus >0.1 U/kg SC insulin administered every two hours). This review did not identify a significant advantage of SC insulin in the reduction in hypoglycemia, hypokalemia, or other outcomes between the two approaches [22,30,36]. Similarly, no significant differences were observed when comparing continuous IV insulin infusion with intermittent SC insulin administration every two hours, although the available evidence was limited and of low certainty. Physiologically, in mild-to-moderate DKA where perfusion is preserved, SC absorption of insulin remains reliable. However, in more severe DKA or shock, poor peripheral perfusion may impair SC absorption and bioavailability, favoring IV routes. One physiological explanation for this finding is that the pharmacokinetics of rapid-acting SC insulin analogs (e.g., lispro and aspart) approximate the continuous availability of insulin achieved by IV infusion when given at sufficiently frequent intervals. Peak plasma levels of SC analogs occur typically around 60 min, and frequent administration (e.g., hourly) can maintain relatively steady plasma insulin levels [35,37].

These findings are consistent with current guidelines, which favor IV insulin for severe DKA due to its rapid onset and titratability but recognize SC insulin as an alternative in mild cases or when IV access is challenging [12,35]. For example, ISPAD explicitly notes that hourly or bi-hourly SC analog dosing “is safe and may be as effective” as IV infusion in stable children [35]. The Texas Children’s DKA guideline also recommends IV insulin as preferred but concedes that serial SC injections (every 2 to 4 h) are “safe and may be as effective” in cases of uncomplicated DKA [38].

Similar to our findings of no significant differences between IV and SC routes, existing reviews found limited trials addressing route or frequency, leading to wide confidence intervals around those comparisons [4]. The lack of significant differences between IV and SC routes, coupled with the potential for frequent SC rapid-acting insulin administration to achieve steady levels, suggests that SC insulin could be a valuable alternative, particularly in resource-limited settings. In such contexts, where the burden of diabetes is high, and continuous IV infusions may be logistically challenging or costly, the programmatic implementation and safety of using SC insulin are critical considerations.

This review highlights critical gaps in the literature. The very low certainty of evidence for most outcomes reflects the methodological limitations of included trials, such as open-label designs, small sample sizes, and inconsistent diagnostic criteria for DKA-related complications. Additionally, the low incidence of cerebral injury limits the ability to draw definitive conclusions about the protective effects of specific regimens, and the absence of mortality restricts insights into survival benefits.

No RCT had rigorously compared IV versus SC insulin in severe pediatric DKA, or continuous versus intermittent regimens in a broad population. A critical gap thus remains in robust comparative trials for IV versus SC insulin or continuous versus intermittent regimens, particularly across the full spectrum of DKA severity. Notably, most included studies focused on mild-to-moderate DKA, and our conclusions therefore apply to this subgroup. Severe DKA requires separate evaluations, such as insulin resistance, perfusion deficits and metabolic instability, to alter treatment response. Existing reviews corroborate the limited and low-quality data on SC protocols [4], but this is inconclusive evidence of equivalence for route or frequency.

To strengthen the evidence base, future high-quality, multicenter RCTs are essential. These trials must be adequately powered to assess serious outcomes such as cerebral edema and mortality, to confirm that altering the dose or route does not inadvertently increase risk. Moreover, core outcomes set with standardized definitions (e.g., uniform pH and bicarbonate thresholds for DKA severity, clear criteria for hypoglycemia) are needed to reduce heterogeneity and improve comparability. Pragmatic trials assessing the feasibility, safety, and cost-effectiveness of SC insulin are also urgently needed, especially in resource-constrained environments where its programmatic implementation holds significant potential.

### Implications for Practice

This review suggests that the use of low-dose insulin (0.05 U/kg/h) is appropriate for selected patients with mild-to-moderate DKA, as it reduces the risk of iatrogenic complications such as hypoglycemia and hypokalemia without compromising treatment efficacy. However, this dose may not be suitable for severe DKA, particularly in insulin-resistant adolescents, obese individuals, or those with concurrent sepsis. In resource-limited or non-critical settings, SC insulin analogs offer a viable alternative to IV infusion for mild DKA cases. Nonetheless, the overall certainty of evidence for route and frequency of insulin administration is low; hence, the results remain inconclusive. Until stronger evidence becomes available, current practice should continue to be guided by existing research and expert consensus.

## 5. Conclusions

This systematic review suggests the safety and efficacy of lower insulin doses in mild-to-moderate pediatric DKA, with significant reductions in hypoglycemia and hypokalemia. These findings offer valuable guidance for clinicians while also emphasizing the need for further high-quality research to validate these results, refine treatment protocols and improve outcomes for children and adolescents with DKA.

## Figures and Tables

**Table 1:** Inclusion and exclusion criteria.

Inclusion Criteria	
Population: Children and adolescents with a confirmed diagnosis of DKA, with or without signs of shock, age 1–19 years, and with T1DM only	
Setting: Low-, middle-, and high-income countries.	
Study design: Randomized Controlled Trials	
Type of Interventions:	
	Insulin administered through IV infusion Frequency of insulin delivery every 4th h Insulin dose 0.05 U/kg/h
Subgroup dosing with rapid-acting vs. short-acting	
Types of comparator/control:	
	Insulin administered through IM, SC, or other routes. Other time intervals for insulin administration. IInsulin doses of 1.0 U/kg/h
Exclusion criteria	
Population:	
	Diagnosed with syndromic diabetes, neonatal diabetes, corticosteroid-induced diabetes, maturity-onset diabetes of the young, and gestational diabetes Adults Animal studies
Study design: Case reports, case series, cross-sectional, case–control, cohort studies, opinions, editorials, conference abstracts, reviews, and systematic reviews.	
	Studies that do not report clinical outcomes (e.g., morbidity, mortality, adverse events, and length of hospital stay). Studies including only highly selected groups—such as children being treated with high-dose corticosteroids or receiving chemotherapy were excluded.

**Table 2:** Study Characteristics.

Study	Country	Blinding	Age Range	Sample Size	Gender Intervention (%)	Gender Control (%)	Outcomes Reported
[Burghen, 1980] [21]	USA	Open label	6.2 Y–15.8 Y	32	N/A		Morbidity (hypoglycemia, hypokalemia), mortality
[Saffari, 2024] [31]	Iran	Open label	2 Y–15 Y	108	M = 41.7	M = 58.3	Morbidity (cerebral edema, hypoglycemia, hypokalemia), hospital stay
					F= 58.3	F = 41.7	
[Della Manna, 2005] [22]	Brazil	Open label	3 Y–18 Y	60	M = 32	M = 23.8 F = 76.2	Morbidity (cerebral edema, hypoglycemia, hypokalemia), mortality, adverse events
					F = 68		
[Drop, 1977] [23]	USA	Open label	5 Y–17 Y	14	N/A		Morbidity (hypoglycemia, hypokalemia)
[Lindsay, 1989] [25]	USA	Open label	2 Y–17 Y	38	N/A		Morbidity (cerebral edema)
[Nallasamy, 2014] [26]	India	Open label	0 Y–12 Y	50	M = 36	M = 44	Morbidity (cerebral edema, hypoglycemia, hypokalemia), mortality
					F = 64	F = 56	
[Onur, 1979] [27]	USA	Open label	4 Y–15 Y	10	N/A		Morbidity (cerebral edema, hypoglycemia, hypokalemia), adverse events
[Perkin, 1979] [28]	USA	Open label	N/A	58	N/A		Morbidity (hypoglycemia, hypokalemia)
[Rameshkumar, 2021] [29]	India	Double blinding	0 Y–12 Y	60	N/A		Morbidity (cerebral edema, hypoglycemia, hypokalemia), mortality
[Razavi, 2018] [30]	Iran	Open label	2 Y–17 Y	50	M = 52	M = 36	Mortality, Hospital stay, Adverse Events
					F = 48	F = 64	
[Saikia, 2022] [32]	India	Open label	0 Y–12 Y	30	M = 46.7 F = 53.3	M = 26.7 F = 73.3	Morbidity (cerebral edema, hypoglycemia, hypokalemia), mortality

## Supplementary Materials

The following supporting information can be downloaded at: https://www.mdpi.com/article/10.3390/jcm14217792/s1.
