# Supplementary material for: Systematic Review and Meta-Analysis of Insulin Dose and Route of Administration Regimens for Diabetic Ketoacidosis in Children and Adolescents
Source: J Clin Med. 2025 Nov 3;14(21):7792. doi: 10.3390/jcm14217792 (PMC12608122; doi:10.3390/jcm14217792)
Supplement: Supplementary file 1 [file jcm-14-07792-s001.zip › jcm-3891184-supplementary.pdf]

SUPPLEMENTARY FILE

Systematic Review and Meta-analysis of Insulin Dose and Route of Administration Regimens for Diabetic Ketoacidosis in Children and Adolescents

Supplementary S1: Search Strategy

PubMed

(Child [MeSH] OR child* OR Pediatrics [MeSH] OR toddler* OR kids OR kid OR juvenile OR pediatric* OR paediatric* OR preschool OR Adolescent [MeSH] OR adolescent [tiab] OR Adolescence OR Teen* OR Youth OR Youths) AND ("diabetic keto*" OR DKA OR "diabetic ketoacidosis" [MeSH] OR "diabetic ketoacidosis" [tiab] OR "diabetic ketoacidoses" OR "diabetic ketosis") AND (insulin OR "insulin administration" OR "insulin therapy" OR "insulin infusion" OR "insulin delivery" OR "insulin regimen" OR "parenteral infusion" OR "subcutaneous infusion" OR "intravenous infusion" OR "short-acting insulin" OR "rapid-acting insulin" OR "long-acting insulin" OR "fast-acting insulin" OR "basal insulin" OR "bolus insulin" OR "low-dose insulin" OR "standard-dose insulin" OR "insulin pump" OR "insulin pumps" OR "insulin pen" OR "insulin syringe" OR "insulin syringes" OR "insulin injection" OR "insulin injections" OR "insulin aspart" OR "insulin lispro" OR "insulin glulisine" OR "insulin detemir" OR "insulin glargine" OR "insulin degludec") AND ("Randomized Controlled Trial" [Publication Type] OR "Randomized Controlled Trials as Topic"[Mesh] OR random* OR "RCT" OR trial)
Filters: Humans, English

CINAHL

((Child* OR Pediatric* OR toddler* OR kids OR kid OR juvenile OR paediatric* OR preschool OR adolescent OR Adolescence OR Teen* OR Youth OR Youths)) AND (("diabetic keto*" OR DKA OR "diabetic ketoacidosis" OR "diabetic ketoacidosis" [tiab] OR "diabetic ketoacidoses" OR "diabetic ketosis")) AND ((insulin OR “insulin administration” OR “insulin therapy” OR “insulin infusion” OR “insulin delivery” OR “insulin regimen” OR “parenteral infusion” OR “subcutaneous infusion” OR “intravenous infusion” OR “short*acting insulin” OR “rapid*acting insulin” OR “long*acting insulin” OR “fast*acting insulin” OR “basal insulin” OR “bolus insulin” OR “low*dose insulin” OR “standard*dose insulin” OR “insulin pump” OR “insulin pumps” OR “insulin pen” OR “insulin syringe” OR “insulin syringes” OR “insulin injection” OR “insulin injections” OR “insulin aspart” OR “insulin lispro” OR “insulin glulisine” OR “insulin detemir” OR “insulin glargine” OR “insulin degludec”)) AND ((("Random Assignment") OR RANDOM* OR RCT OR trial OR ("Randomized Controlled Trials*")))
Limiters - English Language; Research Article; Human; Randomized Controlled Trials; Language: English; Age Groups: Child, Preschool: 2-5 years, Child: 6-12 years, Adolescent: 13-18 years, All Child

Cochrane

ID Search

#1 MeSH descriptor: [Child] explode all trees

#2 MeSH descriptor: [Pediatrics] explode all trees

#3 MeSH descriptor: [Adolescent] explode all trees

#4 (child* OR pediatric* OR toddler* OR kids OR kid OR juvenile OR paediatric* OR preschool OR adolescent OR Adolescence OR Teen* OR Youth OR Youths):ti,ab,kw

#5 MeSH descriptor: [Diabetic Ketoacidosis] explode all trees

#6 ((diabetic NEXT ketoacidosis) OR DKA OR "diabetic ketosis"):ti,ab,kw

#7 (insulin OR “insulin administration” OR “insulin therapy” OR “insulin infusion” OR “insulin delivery” OR “insulin regimen” OR “parenteral infusion” OR “subcutaneous infusion” OR “intravenous infusion” OR “short-acting insulin” OR “rapid-acting insulin” OR “long-acting insulin” OR “fast-acting insulin” OR “basal insulin” OR “bolus insulin” OR “low-dose insulin” OR “standard-dose insulin” OR “insulin pump” OR “insulin pumps” OR “insulin pen” OR “insulin syringe” OR “insulin syringes” OR “insulin injection” OR “insulin injections” OR “insulin aspart” OR “insulin lispro” OR “insulin glulisine” OR “insulin detemir” OR “insulin glargine” OR “insulin degludec”):ti,ab,kw

#8 MeSH descriptor: [Randomized Controlled Trial] explode all trees

#9 MeSH descriptor: [Randomized Controlled Trials as Topic] explode all trees

#10 (random* OR RCT OR trial):ti,ab,kw

#11 #1 OR #2 OR #3 OR #4

#12 #5 OR #6

#13 #8 OR #9 OR #10

#14 #11 AND #12 AND #7 AND #13

Scopus

(TITLE-ABS-KEY (child* OR pediatric* OR toddler* OR kids OR kid OR juvenile OR paediatric* OR preschool OR “adolescent” [tiab] OR adolescence OR teen* OR youth OR youths) AND TITLE-ABS-KEY ("diabetic keto*" OR dka OR "diabetic ketoacidosis" OR "diabetic ketoacidosis" [tiab] OR "diabetic ketoacidoses" OR "diabetic ketosis") AND TITLE-ABS-KEY (insulin OR "insulin administration" OR "insulin therapy" OR "insulin infusion" OR "insulin delivery" OR "insulin regimen" OR "parenteral infusion" OR "subcutaneous infusion" OR "intravenous infusion" OR "short-acting insulin" OR "rapid-acting insulin" OR "long-acting insulin" OR "fast-acting insulin" OR "basal insulin" OR "bolus insulin" OR "low-dose insulin" OR "standard-dose insulin" OR "insulin pump" OR "insulin pumps" OR "insulin pen" OR "insulin syringe" OR "insulin syringes" OR "insulin injection" OR "insulin injections" OR "insulin aspart" OR "insulin lispro" OR "insulin glulisine" OR "insulin detemir" OR "insulin glargine" OR "insulin degludec") AND (random* OR "RCT" OR trial)) AND (LIMIT-TO (DOCTYPE , "ar")) AND (EXCLUDE (SUBJAREA , "VETE")) AND (LIMIT-TO (LANGUAGE , "English")) AND (LIMIT-TO (EXACTKEYWORD , "Human") OR EXCLUDE (EXACTKEYWORD , "Animals") OR EXCLUDE (EXACTKEYWORD , "Animal"))

Clinicaltrials.gov

“Diabetic Ketoacidosis” OR "diabetic keto*" OR DKA OR "diabetic ketoacidoses" OR "diabetic ketosis" | Other terms: Child* OR Pediatric* OR toddler* OR kids OR kid OR juvenile OR paediatric* OR preschool OR adolescent OR Adolescence OR Teen* OR Youth OR Youths | (insulin OR “insulin administration” OR “insulin therapy” OR “insulin infusion” OR “insulin delivery” OR “insulin regimen” OR “parenteral infusion” OR “subcutaneous infusion” OR “intravenous infusion” OR “short-acting insulin” OR “rapid-acting insulin” OR “long-acting insulin” OR “fast-acting insulin” OR “basal insulin” OR “bolus insulin” OR “low-dose insulin” OR “standard-dose insulin” OR “insulin pump” OR “insulin pumps” OR “insulin pen” OR “insulin syringe” OR “insulin syringes” OR “insulin injection” OR “insulin injections” OR “insulin aspart” OR “insulin lispro” OR “insulin glulisine” OR “insulin detemir” OR “insulin glargine” OR “insulin degludec”) | Interventional studies

ICTRP

(child* OR toddler* OR kids OR kid OR juvenile OR pediatric* OR paediatric* OR preschool OR Adolescent OR Adolescence OR Teen* OR Youth OR Youths) AND ("diabetic keto*" OR DKA OR "diabetic ketoacidosis" OR "diabetic ketoacidoses" OR "diabetic ketosis") AND (insulin OR "insulin administration" OR "insulin therapy" OR "insulin infusion" OR "insulin delivery" OR "insulin regimen" OR "parenteral infusion" OR "subcutaneous infusion" OR "intravenous infusion" OR "short-acting insulin" OR "rapid-acting insulin" OR "long-acting insulin" OR "fast-acting insulin" OR "basal insulin" OR "bolus insulin" OR "low-dose insulin" OR "standard-dose insulin" OR "insulin pump" OR "insulin pumps" OR "insulin pen" OR "insulin syringe" OR "insulin syringes" OR "insulin injection" OR "insulin injections" OR "insulin aspart" OR "insulin lispro" OR "insulin glulisine" OR "insulin detemir" OR "insulin glargine" OR "insulin degludec”)

Supplementary S2: Insulin Regimens Administered

Study	Dose, Route and Type of Insulin – Intervention	Dose, Route and Type of Insulin – Control	Insulin route, frequency and dose after resolution of DKA	Biochemical criteria for resolution of DKA	Glucose measured	Bolus dose of fluid (rate-type-duration)	Maintenance dose of fluid f(rate-type-duration)
**Burghen (1980)**	0.1 U/kg/h IV Crystalline Insulin	1.0 U/kg/h IV Crystalline Insulin	• In first 2 hours if decrease by 10% IV push 0.2 U/kg in low dose and 1.0 U/kg in high dose.<br>• If plasma glucose < 250 mg/dL insulin reduced to 0.5 U/kg/h in high dose.<br>• If plasma glucose < 100 mg/dL insulin discontinued in both groups	Glucose to reach 250 mg/dL, plasma bicarbonate to reach 15 meq/L, arterial blood pH to reach 7.30, and plasma ketones to be negative at a dilution of 1:2	Hourly	If the arterial blood pH was less than 7.0, additional NaHCO3 (1 mM/kg) was infused during the first 15 min of therapy; this amount was repeated if the pH was not increased to 7.1 or above in 2 h.	During the first 2 h of treatment, a solution containing 75 mM sodium chloride and 50 mM NaHCO3, 500 mg of albumin per liter and insulin was infused at a rate of 200 ml/ m2 body surface area per hour.
**Saffari (2024)**	0.05 - 0.1 U/kg/h of regular insulin given IV, followed by 0.5U/kg of insulin Detemir	0.05 - 0.1 U/kg/h of regular insulin given IV	N/A	N/A	Hourly	Isotonic (0.9%) saline was used as rehydrating fluid for at least the initial 6 hours	Half normal (0.45%) saline. Dextrose (5%) was added once the BG level

	given SC **(Group 1)** and Glargine **(Group 2)** for 24 hours if needed						decreased to 250 mg/dL or less and then to maintain a BG level between 180 and 220 mg/dL. Potassium chloride (40 mEq/L) to maintain a serum potassium level between 3.5 and 5.5 mEq/L
**Della Manna (2005)**	0.1 U/kg/ Regular IV given continuously for 12hrs until capillary blood glucose levels neared 13.8 mmol/l	0.15 U/kg/h SC Lispro given every 2 hours until capillary blood glucose levels neared 13.8 mmol/l	After 12h of intensive fast-acting insulin administration, intermediate human insulin was initiated at a dosage of 0.4 unit/kg every 12 h in both groups every 4 hour for the next 24 hours	0.15 U/kg given Subcutaneously for 30 mins before stopping the intravenous line. Intermediate human insulin was initiated at a dosage of 0.4 unit/kg every 12 h in both groups every 4 hours for the next 24 hours	Electrolytes (ionselective electrode) and plasma blood glucose (ultraviolet hexokinase), were measured on admission, 2 h after admission, at the time capillary blood glucose	Normal saline (0.9% NaCl) was infused at the rate of 20 ml/kg/hr to restore peripheral perfusion till the 2nd hr	After the 2nd h, the infusion rate of normal saline was decreased to 10 ml/kg/hr until volume reparation was achieved.

					reached 13.8 mmol/l, and 6, 12, and 24 h thereafter		
**Drop (1977)**	0.1 U/kg/h of IV crystalline insulin (0.5 ml of Iletin) given until BG fallen to 200 to 250 mg/100 ml	One third of the dose 0.9-1.8 U/kg was given as an intravenous bolus and the rest was given subcutaneously until BG fallen to 200 to 250 mg/100 ml	0.1-0.25 U/kg/h SC given for 2-6 hours according to results of blood and urine glucose and ketone levels	0.1-0.25 U/kg/h SC 2 – 6 hours according to results of blood and urine glucose and ketone levels	Blood glucose levels estimated every 30 minutes. and serum glucose and ketone levels determined hourly.	Isotonic saline at a rate of 360 mI/sq m of body surface area given over 30 to 45 minutes	Followed by isotonic saline at rate of 270 mI/sq ni/hr over the next three hours 0.45% saline was given in either 2.5% or 5% dextrose in water, the latter as the blood glucose level approached 250 mg/mI during the second three hours, 0.23% saline in 5% dextrose (Normosol) was infused at a rate of 150 nil/sq rn/hr during the six to

							eight hours after admission,
**Lindsay (1989)**	0.1 U/kg IV bolus given once and then switched to continuous infusion as mentioned later on. Once bolus ended, shifted to maintenance	N/A	0.1 U/kg/h Continuous IV infusion IV insulin infusion was discontinued when the acidosis had resolved (serum bicarbonate level ≥17 mEq/L), and the acetonemia was reduced to a level of ≤20 mg/dl.	0.1 U/kg/h Continuous IV infusion<br>IV insulin infusion was discontinued when the acidosis had resolved (serum bicarbonate level ≥17 mEq/L), and the acetonemia was reduced to a level of ≤20 mg/dl.	N/A for glucose. electrolytes drawn one hr after the start of insulin and then every 4-6hrs	20 ml/kg intravenous bolus of 0.9% saline over 30 to 45 minutes. Children who appeared severely dehydrated were then given a second 10 to 20 ml/kg bolus of 0.9% saline. A second serum glucose level was drawn at the completion of the fluid bolus. The insulin bolus group was then given	At the completion of the 0.9% saline bolus, each group was given 0.45% saline with 20 mEq/L of potassium acetate and 20 mEq/L of potassium phosphate at 1.5 times maintenance rate. Ten percent dextrose was added to the solution when the serum glucose level was ≤250 mg/dl.
**Nallasmy (2014)**	0.05 U/kg/h IV regular insulin	0.1 U/kg/h IV regular insulin	SC regular insulin every 30 minutes	Correction of acidosis (pH >= 7.3 and	Hourly or more frequently	Isotonic (0.9%) saline was used as	Half normal (0.45%) saline.

				bicarbonate >=15 mEq/L) was taken as end point.		rehydrating fluid for at least the initial 6 hours	Dextrose (5%) was added once the Blood Glucose (BG) level decreased to 250 mg/dL or less and then to maintain a BG level between 180 and 220 mg/dL. Potassium chloride (40 mEq/L) to maintain a serum potassium level between 3.5 and 5.5 mEq/L
**Onur (1979)**	N/A	N/A	0.1 U/kg IM Regular insulin every 2 hours until their serum glucose concentrations reached 250 mg/dl or less.	1.0 U/kg regular insulin.	Blood samples for glucose, electrolytes, and hormone assays were obtained every two hours until the serum glucose	0.9% NaCl, K + (30mEq/l) and HPO4=-H2PO4 infusion at a rate of 20 ml/kg/hour for the first two hours	At the end of this period, fluids were given as 0.45% NaC1 with K + and HPO4=-H~PO, at a rate to correct
			When serum glucose	Half of the calculated dose			

			concentration was 250 mg or less, fractional urine coverage with regular insulin was started.	was given subcutaneously, the other half intravenously by push every 4 hours until their serum glucose concentrations reached 250 mg/dl or less.	concentrations were 250 mg/dl or less. Dextrostix estimations for serum glucose were carried out every two hours.		estimated deficits, to provide maintenance and to replace excessive ongoing losses. Half the calculated amount was given during the first eight hours and the other half in the remaining 16 hours. After the serum glucose concentration reached 250 mg/dl or less, fluids were given as 5% dextrose and 0.45% NaCI with K" and HPO4 = - H2PO4
**Perkin (1979)**	0.25 U/kg Regular IV	2 U/kg Regular insulin An additional	0.1 U/kg/h IV continuous regular insulin. The infusion	1 U/kg SC regular insulin every 2 hours	Every two hours until the	Non-glucose-containing fluids were	Followed by a mixture of 60% D10W

	given once plus additional dose	1U/kg was administered IV at one hour if the admission blood glucose was greater than 500mg/dI.<br><br>One-half the dose was administered intravenously and one-half subcutaneously.	was continued in each patient until the serum acetone was trace or negative at which time the transition was made	until the serum acetone had cleared. Time transition was made to a sliding scale subcutaneous administration. Because of the short intravenous half-life of insulin, an overlap period of 30 minutes was used in the transition.	correction of ketoacidosis	administered for the first two hours	and 40% Ringer's lactate with 40 mEq K+/200 ml of fluid. Rates were calculated to give 1.5 times maintenance requirements plus replacement for 8 to 10 per cent dehydration.
**Rameshkumar (2021)**	0.05 U/kg/h IV regular insulin	0.1 U/kg/h IV regular insulin	Shifted to regular subcutaneous insulin with an overlap time of 30 minutes with intravenous insulin.	Resolution of ketoacidosis (pH ≥7.30, bicarbonate ≥15 mEq/L, and BOHB <1 mmol/L)	Every half hourly	20 mL/kg of normal saline.	Normal saline was used for the first six hours and was changed to 0.45% saline. Dextrose (5%) was added once the blood glucose level decreased to 250 mg/dL or less. Potassium

							chloride (40 mEq/L) was added to maintain a serum level of 3.5-5.5 mEq/L
						Children with shock received an additional 20 mL/kg of normal saline for one hour	
**Razavi (2018)**	N/A	N/A	0.05-0.1 U/kg/h starting dose of IV insulin administered in children with moderate DKA was 0.1 units/kg/hour and for children with mild DKA was 0.05 units/kg/hour until resolution of DKA	0.15 U/kg SC Aspart given every 2 hours until resolution of DKA	Bedside capillary blood glucose levels, as well as venous blood gas and electrolytes were measured every 2 h and then every 4 h until resolution of diabetic ketoacidosis.	Normal saline (0.9% NaCl) was infused at the rate of 10–20 cc/kg within an hour as initial fluid resuscitation in patients with dehydration. Patients with persistently poor perfusion received additional infusions of	Subsequent fluid replacement was administered as half saline according to Milwaukee DKA protocol (IV rate = 85 mL/kg + maintenance − bolus/23 h). Initial bolus therapy with normal saline (20 cc/kg)

						0.9% saline until hemodynamic stability was achieved	was subtracted from volume deficit.
**Saikia (2022)**	0.05 U/kg/h IV regular insulin	0.1 U/kg/h IV regular insulin	Insulin was gradually tapered, and subcutaneous insulin was initiated with overlap periods as per the type of insulin.	Resolution of acidosis (pH ≥7.3 and HCO3 ≥15).	Hourly	0.9% Normal Saline	0.9% Normal Saline + 0.45%. 5% dextrose was added

Supplementary S3: RoB-2 Assessment

Supplementary S3 Figure S1: Cerebral Injury (n=8)

Study	D1	D2	D3	D4	D5	Overall
DellaManna 2005	!	!	+	!	!	!
Edwards 1977	!	+	+	-	!	-
Lindsay 1989	-	!	+	!	!	-
Nallasamy 2014	+	+	+	-	!	-
Onur 1979	!	!	+	!	!	!
Rameshkumar 2021	+	+	+	+	!	!
Saffari 2024	!	!	+	!	!	!
Saikia 2022	+	!	+	!	!	!

D1: Randomisation process
D2: Deviations from the intended interventions
D3: Missing outcome data
D4: Measurement of the outcome
D5: Selection of the reported result

Supplementary S3 Figure S2: Hypoglycemia (n=10)

Study	D1	D2	D3	D4	D5	Overall
Burghen 1980	!	!	+	+	!	!
DellaManna 2005	!	!	+	+	!	!
Drop 1977	-	!	+	+	!	-
Edwards 1977	!	+	+	!	!	!
Nallasamy 2014	+	+	+	+	+	+
Onur 1979	!	!	+	+	!	!
Perkin 1979	!	+	+	+	!	!
Rameshkumar 2021	+	+	+	+	+	+
Saffari 2024	!	!	+	!	!	!
Saikia 2022	+	!	+	!	!	!

D1: Randomisation process
D2: Deviations from the intended interventions
D3: Missing outcome data
D4: Measurement of the outcome
D5: Selection of the reported result

Supplementary S3 Figure S3: Hypokalemia (n=10)

Study	D1	D2	D3	D4	D5	Overall
Burghen 1980	!	!	+	+	!	!
DellaManna 2005	!	!	+	+	!	!
Drop 1977	-	!	+	+	!	-
Edwards 1977	!	+	+	+	!	!
Nallasamy 2014	+	+	+	+	+	+
Onur 1979	!	!	+	+	!	!
Perkin 1979	-	+	+	+	!	-
Rameshkumar 2021	+	+	+	+	+	+
Saffari 2024	!	!	+	+	!	!
Saikia 2022	+	!	+	+	!	!

D1: Randomisation process
D2: Deviations from the intended interventions
D3: Missing outcome data
D4: Measurement of the outcome
D5: Selection of the reported result

Supplementary S3 Figure S4: Mortality (n=6)

Study	D1	D2	D3	D4	D5	Overall
Burghen 1980	!	!	+	+	!	!
DellaManna 2005	!	!	+	+	!	!
Nallasamy 2014	+	+	+	+	!	!
Rameshkumar 2021	+	+	+	+	+	+
Razavi 2018	!	!	+	+	!	!
Saikia 2022	+	!	+	+	!	!

D1: Randomisation process
D2: Deviations from the intended interventions
D3: Missing outcome data
D4: Measurement of the outcome
D5: Selection of the reported result

Supplementary S3 Figure S5: Hospital Stay (n=2)

Study	D1	D2	D3	D4	D5	Overall
Razavi 2018	!	!	+	!	!	!
Saffari 2024	!	!	-	-	!	-

D1: Randomisation process
D2: Deviations from the intended interventions
D3: Missing outcome data
D4: Measurement of the outcome
D5: Selection of the reported result

Supplementary S3 Figure S6: Adverse Events (n=3)

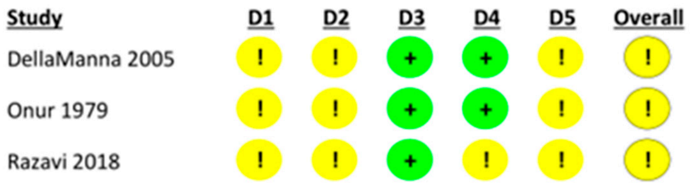

D1: Randomisation process
D2: Deviations from the intended interventions
D3: Missing outcome data
D4: Measurement of the outcome
D5: Selection of the reported result

Supplementary S4: Forest Plots for all Comparisons

Supplementary S4 Figure S1: Dose: 0.05 U/kg/h IV insulin vs 0.1 U/kg/h IV insulin

Study or Subgroup	0.05 U/kg/h IV Insulin Events	0.05 U/kg/h IV Insulin Total	0.1 U/kg/h IV Insulin Events	0.1 U/kg/h IV Insulin Total	Weight	Risk Ratio M-H, Random, 95% CI
Nallasamy 2014 (1)	0	25	1	25	100.0%	0.33 [0.01, 7.81]
Rameshkumar 2021 (2)	0	30	0	30		Not estimable
Saikia 2022 (3)	0	15	0	15		Not estimable
**Total (95% CI)**		**70**		**70**	**100.0%**	**0.33 [0.01, 7.81]**
Total events	0		1			

Heterogeneity: Not applicable
Test for overall effect: Z = 0.68 (P = 0.49)

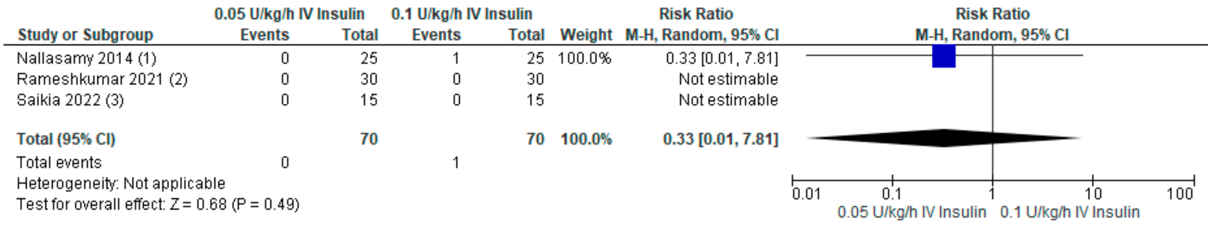

Footnotes
(1) Regular (short-acting insulin)
(2) Regular (short-acting insulin)
(3) Regular (short-acting insulin)

Supplementary S4 Figure S1.1: Forest plot for outcome = Morbidity: Cerebral injury (n=3)

Study or Subgroup	0.05 U/kg/h IV Insulin Events	0.05 U/kg/h IV Insulin Total	0.1 U/kg/h IV Insulin Events	0.1 U/kg/h IV Insulin Total	Weight	Risk Ratio M-H, Random, 95% CI
Nallasamy 2014 (1)	1	25	5	25	15.1%	0.20 [0.03, 1.59]
Rameshkumar 2021 (2)	1	30	4	15	14.7%	0.13 [0.02, 1.02]
Saikia 2022 (3)	4	15	8	15	70.1%	0.50 [0.19, 1.31]
**Total (95% CI)**		**70**		**55**	**100.0%**	**0.35 [0.16, 0.80]**
Total events	6		17			

Heterogeneity: $Tau^2 = 0.00$; $Chi^2 = 1.84$, df = 2 (P = 0.40); $I^2 = 0\%$
Test for overall effect: Z = 2.52 (P = 0.01)

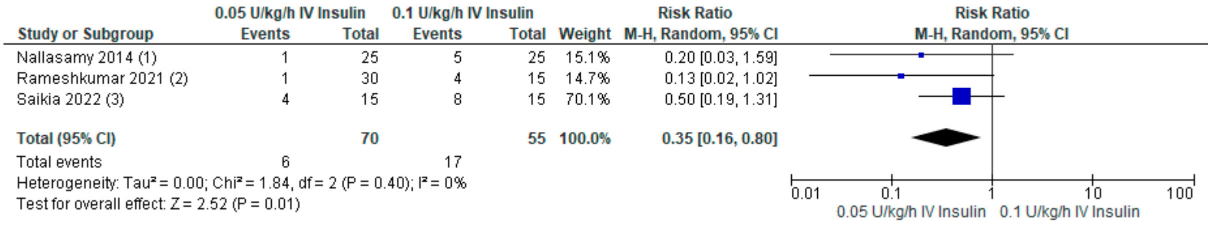

Footnotes
(1) Regular (short-acting insulin)
(2) Regular (short-acting insulin)
(3) Regular (short-acting insulin)

Supplementary S4 Figure S1.2: Forest plot for outcome = Morbidity: Hypoglycaemia (n=3)

Study or Subgroup	0.05 U/kg/h IV Insulin Events	0.05 U/kg/h IV Insulin Total	0.1 U/kg/h IV Insulin Events	0.1 U/kg/h IV Insulin Total	Weight	Risk Ratio M-H, Random, 95% CI
Nallasamy 2014 (1)	5	25	12	25	30.6%	0.42 [0.17, 1.01]
Rameshkumar 2021 (2)	9	30	13	30	51.3%	0.69 [0.35, 1.37]
Saikia 2022 (3)	3	15	7	15	18.1%	0.43 [0.14, 1.35]
**Total (95% CI)**		**70**		**70**	**100.0%**	**0.54 [0.33, 0.89]**
Total events	17		32			

Heterogeneity: $Tau^2 = 0.00$; $Chi^2 = 1.00$, df = 2 (P = 0.61); $I^2 = 0\%$
Test for overall effect: Z = 2.45 (P = 0.01)

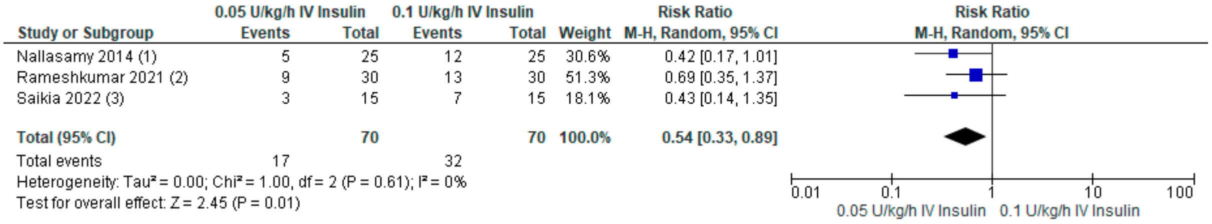

Footnotes
(1) Regular (short-acting insulin)
(2) Regular (short-acting insulin)
(3) Regular (short-acting insulin)

Supplementary S4 Figure S1.3: Forest plot for outcome = Morbidity: Hypokalaemia (n=3)

Study or Subgroup	0.05 U/kg/h IV Insulin Events	0.05 U/kg/h IV Insulin Total	0.1 U/kg/h IV Insulin Events	0.1 U/kg/h IV Insulin Total	Weight	Risk Ratio M-H, Random, 95% CI
Nallasamy 2014 (1)	6	25	18	25	22.2%	0.33 [0.16, 0.70]
Rameshkumar 2021 (2)	10	30	17	30	34.3%	0.59 [0.32, 1.07]
Saikia 2022 (3)	7	15	15	15	43.4%	0.48 [0.29, 0.82]
**Total (95% CI)**		**70**		**70**	**100.0%**	**0.48 [0.34, 0.67]**
Total events	23		50			

Heterogeneity: $Tau^2 = 0.00$; $Chi^2 = 1.40$, df = 2 (P = 0.50); $I^2 = 0\%$
Test for overall effect: Z = 4.17 (P < 0.0001)

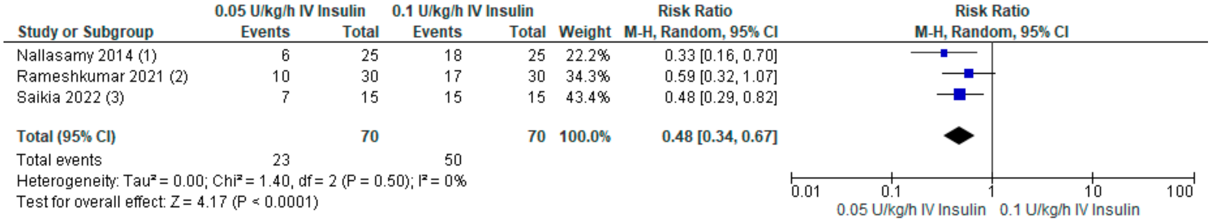

Footnotes
(1) Regular (short-acting insulin)
(2) Regular (short-acting insulin)
(3) Regular (short-acting insulin)

Supplementary S4 Figure S2: Dose: 0.05-0.1 U/kg/h IV Insulin vs >0.1 U/kg/2h SC Insulin

Study or Subgroup	0.05-0.1 U/kg/h IV Insulin Events	Total	>0.1 U/kg/2h SC Insulin Events	Total	Weight	Risk Ratio M-H, Random, 95% CI
DellaManna 2005 (1)	6	30	4	30	100.0%	1.50 [0.47, 4.78]
**Total (95% CI)**		**30**		**30**	**100.0%**	**1.50 [0.47, 4.78]**
Total events	6		4			
Heterogeneity: Not applicable						
Test for overall effect: Z = 0.69 (P = 0.49)						

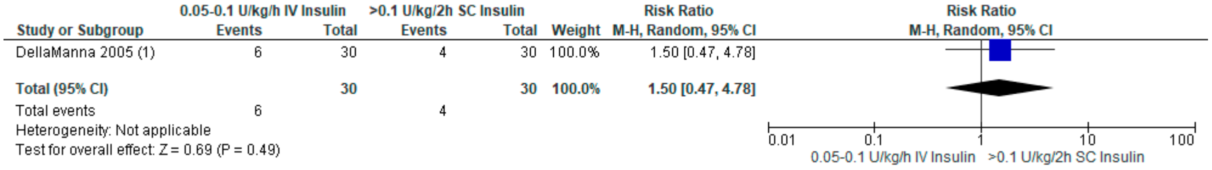

Footnotes
(1) IV = 0.1U/kg/h Regular (Short-acting insulin), SC = 0.15 U/kg Lispro (Fast-acting insulin)

Supplementary S4 Figure S2.1: Forest plot for outcome = Morbidity: Hypoglycaemia

Study or Subgroup	0.05-0.1 U/kg/h IV Insulin Mean [Hours]	SD [Hours]	Total	>0.1 U/kg/2h SC Insulin Mean [Hours]	SD [Hours]	Total	Weight	Mean Difference IV, Random, 95% CI
Razavi 2018 (1)	76.08	9.6	25	79.92	25.68	25	50.4%	-3.84 [-14.59, 6.91]
Razavi 2018 (2)	106.08	26.88	25	81.12	14.4	25	49.6%	24.96 [13.01, 36.91]
**Total (95% CI)**			**50**			**50**	**100.0%**	**10.44 [-17.79, 38.66]**
Heterogeneity: Tau² = 381.09; Chi² = 12.33, df = 1 (P = 0.0004); I² = 92%								
Test for overall effect: Z = 0.72 (P = 0.47)								

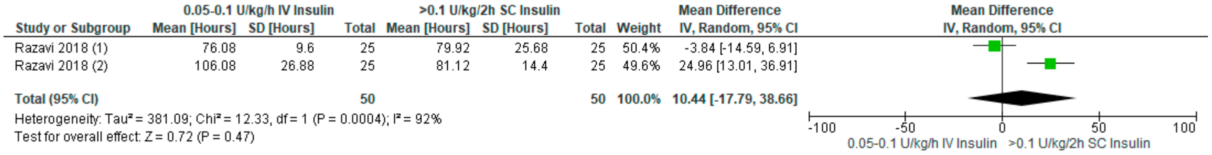

Footnotes
(1) Mild DKA (venous pH <7.3 or bicarbonate <15 mmol/l); IV = Regular (Short-acting insulin), SC = Aspart (Fast-acting insulin)
(2) Moderate DKA (pH <7.2 or bicarbonate <10 mmol/l); IV = Regular (Short-acting insulin), SC = Aspart (Fast-acting insulin)

Supplementary S4 Figure S2.2: Forest plot for outcome = Hospital Stay

Supplementary S4 Figure S3: Route: 0.05-0.1 U/kg/h IV insulin vs >0.1 U/kg SC insulin every 2 hours

Study or Subgroup	Intravenous Insulin (IV) Events	Total	Subcutaneous Insulin (SC) Events	Total	Weight	Risk Ratio M-H, Random, 95% CI
DellaManna 2005 (1)	6	30	4	30	100.0%	1.50 [0.47, 4.78]
**Total (95% CI)**		**30**		**30**	**100.0%**	**1.50 [0.47, 4.78]**
Total events	6		4			
Heterogeneity: Not applicable						
Test for overall effect: Z = 0.69 (P = 0.49)						

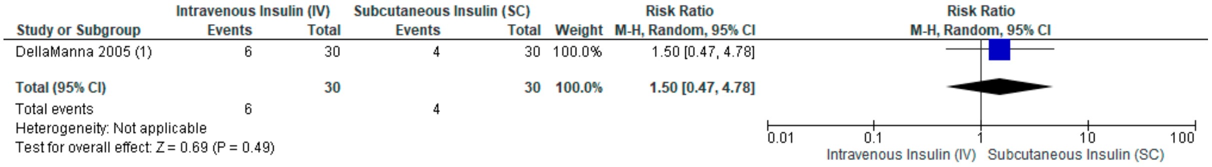

Footnotes
(1) IV = 0.1U/kg/h Regular (Short-acting insulin), SC = 0.15 U/kg Lispro (Fast-acting insulin)

Supplementary S4 Figure S3.1: Forest plot for outcome = Morbidity: Hypoglycaemia

Study or Subgroup	Intravenous Insulin (IV) Mean [Hours]	SD [Hours]	Total	Subcutaneous Insulin (SC) Mean [Hours]	SD [Hours]	Total	Weight	Mean Difference IV, Random, 95% CI
Razavi 2018 (1)	76.08	9.6	25	79.92	25.68	25	50.4%	-3.84 [-14.59, 6.91]
Razavi 2018 (2)	106.08	26.88	25	81.12	14.4	25	49.6%	24.96 [13.01, 36.91]
**Total (95% CI)**			**50**			**50**	**100.0%**	**10.44 [-17.79, 38.66]**
Heterogeneity: Tau² = 381.09; Chi² = 12.33, df = 1 (P = 0.0004); I² = 92%								
Test for overall effect: Z = 0.72 (P = 0.47)								

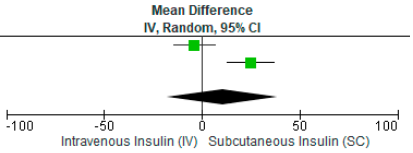

Footnotes
(1) Mild DKA (venous pH <7.3 or bicarbonate <15 mmol/l); IV = Regular (Short-acting insulin), SC = Aspart (Fast-acting insulin)
(2) Moderate DKA (pH <7.2 or bicarbonate <10 mmol/l); IV = Regular (Short-acting insulin), SC = Aspart (Fast-acting insulin)

Supplementary S4 Figure S4: Frequency of Insulin Delivery: Every 2hr vs Continuous Infusion

Study or Subgroup	Every 2hr Events	Every 2hr Total	Continuous Infusion Events	Continuous Infusion Total	Weight	Risk Ratio M-H, Random, 95% CI
DellaManna 2005 (1)	4	30	6	30	100.0%	0.67 [0.21, 2.13]
**Total (95% CI)**		**30**		**30**	**100.0%**	**0.67 [0.21, 2.13]**
Total events	4		6			
Heterogeneity: Not applicable						
Test for overall effect: Z = 0.69 (P = 0.49)						

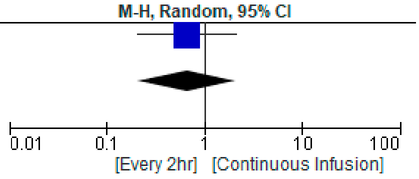

Footnotes
(1) Bolus dose: Every 2hr = 0.1 U/kg/h IV Regular (Short-acting insulin), Continuous infusion = 0.15 U/kg SC Lispro (Fast-acting insulin)

Supplementary S4 Figure S4.1: Forest plot for outcome = Morbidity: Hypoglycaemia

Study or Subgroup	Every 2hr Mean	Every 2hr SD	Every 2hr Total	Continuous Infusion Mean	Continuous Infusion SD	Continuous Infusion Total	Weight	Mean Difference IV, Random, 95% CI
Razavi 2018 (1)	79.92	25.68	25	76.08	9.6	25	50.4%	3.84 [-6.91, 14.59]
Razavi 2018 (2)	81.12	14.4	25	106.08	26.88	25	49.6%	-24.96 [-36.91, -13.01]
**Total (95% CI)**			**50**			**50**	**100.0%**	**-10.44 [-38.66, 17.79]**
Heterogeneity: Tau² = 381.09; Chi² = 12.33, df = 1 (P = 0.0004); I² = 92%								
Test for overall effect: Z = 0.72 (P = 0.47)								

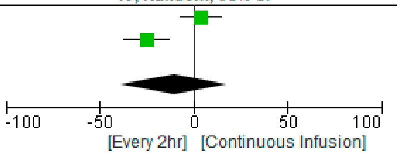

Footnotes
(1) Mild DKA (venous pH <7.3 or bicarbonate <15 mmol/l); Bolus dose: Every 2hr = 0.1 U/kg/h IV Regular (Short-acting insulin), Continuous infusion = 0.15...
(2) Moderate DKA (pH <7.2 or bicarbonate <10 mmol/l); Bolus dose: Every 2hr = 0.1 U/kg/h IV Regular (Short-acting insulin), Continuous infusion = 0.15...

Supplementary S4 Figure S5: Dose: 0.05-0.1 U/kg/h IV + 0.5U/kg SC vs. 0.05-0.1 U/kg/h IV

Study or Subgroup	0.05-0.1 U/kg/h IV + 0.5U/kg SC Events	0.05-0.1 U/kg/h IV + 0.5U/kg SC Total	0.05-0.1 U/kg/h IV Events	0.05-0.1 U/kg/h IV Total	Weight	Risk Ratio M-H, Random, 95% CI
Saffari 2024 (1)	15	72	9	36	100.0%	0.83 [0.40, 1.72]
**Total (95% CI)**		**72**		**36**	**100.0%**	**0.83 [0.40, 1.72]**
Total events	15		9			
Heterogeneity: Not applicable						
Test for overall effect: Z = 0.49 (P = 0.62)						

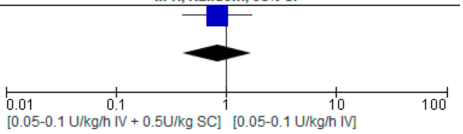

Footnotes
(1) Combination of the Detemir and Glargine arms

Supplementary S4 Figure S5.1: Forest plot for outcome = Morbidity: Cerebral injury

Study or Subgroup	0.05-0.1 U/kg/h IV + 0.5U/kg SC Events	0.05-0.1 U/kg/h IV + 0.5U/kg SC Total	0.05-0.1 U/kg/h IV Events	0.05-0.1 U/kg/h IV Total	Weight	Risk Ratio M-H, Random, 95% CI
Saffari 2024 (1)	11	72	3	36	100.0%	1.83 [0.55, 6.16]
**Total (95% CI)**		**72**		**36**	**100.0%**	**1.83 [0.55, 6.16]**
Total events	11		3			
Heterogeneity: Not applicable						
Test for overall effect: Z = 0.98 (P = 0.33)						

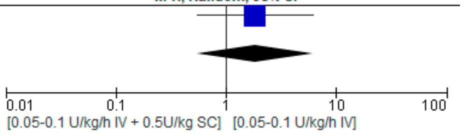

Footnotes
(1) Combination of the Detemir and Glargine arms

Supplementary S4 Figure S5.2: Forest plot for outcome = Morbidity: Hypoglycaemia

Study or Subgroup	0.05-0.1 U/kg/h IV + 0.5U/kg SC Events	0.05-0.1 U/kg/h IV + 0.5U/kg SC Total	0.05-0.1 U/kg/h IV Events	0.05-0.1 U/kg/h IV Total	Weight	Risk Ratio M-H, Random, 95% CI
Saffari 2024 (1)	15	72	7	36	100.0%	1.07 [0.48, 2.39]
**Total (95% CI)**		**72**		**36**	**100.0%**	**1.07 [0.48, 2.39]**
Total events	15		7			
Heterogeneity: Not applicable						
Test for overall effect: Z = 0.17 (P = 0.87)						

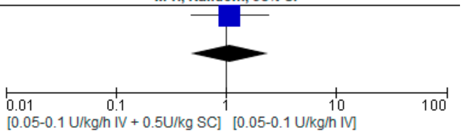

Footnotes
(1) Combination of the Detemir and Glargine arms

Supplementary S4 Figure S5.3: Forest plot for outcome = Morbidity: Hypokalaemia

Study or Subgroup	0.05-0.1 U/kg/h IV + 0.5U/kg SC Events	Total	0.05-0.1 U/kg/h IV Events	Total	Weight	Risk Ratio M-H, Random, 95% CI
Saffari 2024 (1)	41	216	19	108	100.0%	1.08 [0.66, 1.77]
**Total (95% CI)**		**216**		**108**	**100.0%**	**1.08 [0.66, 1.77]**
Total events	41		19			
Heterogeneity: Not applicable						
Test for overall effect: Z = 0.30 (P = 0.76)						

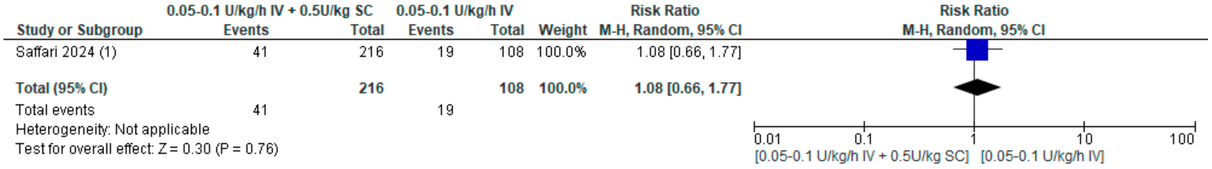

Footnotes
(1) Combination of the Detemir and Glargine arms

Supplementary S4 Figure S6: Dose: 0.1 U/kg/2h IM vs. 1.0 U/kg/4h SC + IV

Study or Subgroup	0.1 U/kg/2h IM Insulin Events	Total	1.0 U/kg/4h SC + IV Insulin Events	Total	Weight	Risk Ratio M-H, Random, 95% CI
Onur 1979	1	7	2	6	100.0%	0.43 [0.05, 3.64]
**Total (95% CI)**		**7**		**6**	**100.0%**	**0.43 [0.05, 3.64]**
Total events	1		2			
Heterogeneity: Not applicable						
Test for overall effect: Z = 0.78 (P = 0.44)						

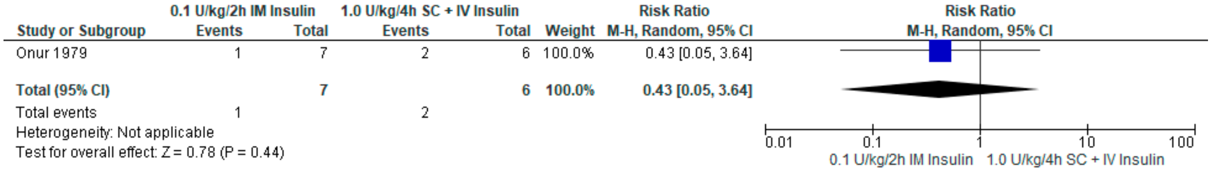

Supplementary S4 Figure S7: Dose: 0.1 U/kg/h IV vs. 1.0 U/kg/h IV

Study or Subgroup	0.1 U/kg/h IV Insulin Events	Total	1.0 U/kg/h IV Insulin Events	Total	Weight	Risk Ratio M-H, Random, 95% CI
Burghen 1980 (1)	2	16	12	16	100.0%	0.17 [0.04, 0.63]
**Total (95% CI)**		**16**		**16**	**100.0%**	**0.17 [0.04, 0.63]**
Total events	2		12			
Heterogeneity: Not applicable						
Test for overall effect: Z = 2.65 (P = 0.008)						

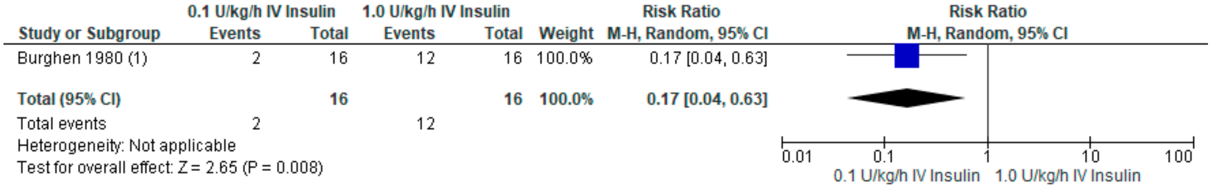

Footnotes
(1) Regular crystalline porcine (short-acting insulin)

Supplementary S4 Figure S7.1: Forest plot for outcome = Morbidity: Hypoglycaemia

Study or Subgroup	0.1 U/kg/h IV Insulin Events	Total	1.0 U/kg/h IV Insulin Events	Total	Weight	Risk Ratio M-H, Random, 95% CI
Burghen 1980 (1)	3	16	10	16	100.0%	0.30 [0.10, 0.89]
**Total (95% CI)**		**16**		**16**	**100.0%**	**0.30 [0.10, 0.89]**
Total events	3		10			
Heterogeneity: Not applicable						
Test for overall effect: Z = 2.17 (P = 0.03)						

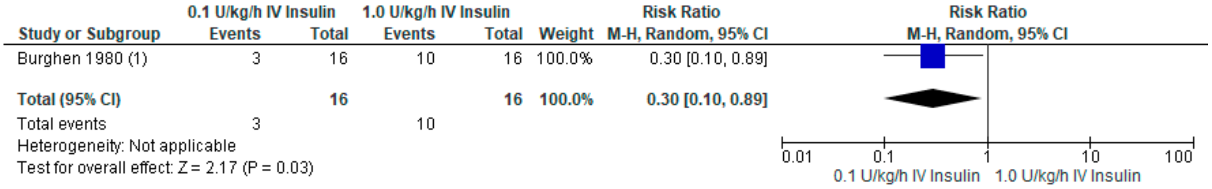

Footnotes
(1) Regular crystalline porcine (short-acting insulin)

Supplementary S4 Figure S7.2: Forest plot for outcome = Morbidity: Hypokalaemia

Study or Subgroup	0.1 U/kg/h IV Insulin Events	Total	1.0 U/kg/h IV Insulin Events	Total	Weight	Risk Ratio M-H, Random, 95% CI
Burghen 1980 (1)	5	32	22	32	100.0%	0.23 [0.10, 0.53]
**Total (95% CI)**		**32**		**32**	**100.0%**	**0.23 [0.10, 0.53]**
Total events	5		22			
Heterogeneity: Not applicable						
Test for overall effect: Z = 3.46 (P = 0.0005)						

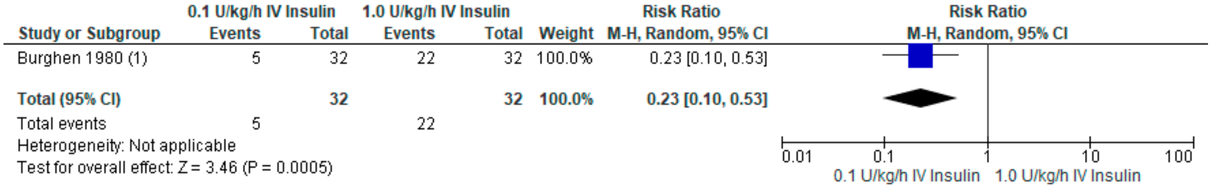

Footnotes
(1) Regular crystalline porcine (short-acting insulin)

Supplementary S4 Figure S8: Dose: 0.1 U/kg/h IV vs. 1.0-2.2 U/kg SC every 3 hours

Study or Subgroup	0.1 U/kg/h IV Events	0.1 U/kg/h IV Total	1.0-2.2 U/kg SC every 3 hours Events	1.0-2.2 U/kg SC every 3 hours Total	Weight	Risk Ratio M-H, Random, 95% CI
Edwards 1977	0	10	1	10	100.0%	0.33 [0.02, 7.32]
**Total (95% CI)**		**10**		**10**	**100.0%**	**0.33 [0.02, 7.32]**
Total events	0		1			

Heterogeneity: Not applicable
Test for overall effect: Z = 0.70 (P = 0.49)

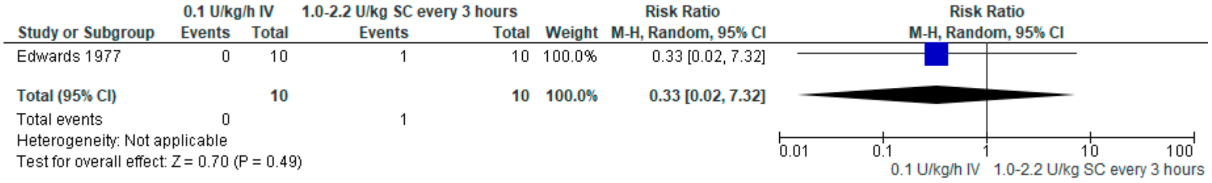

Supplementary S4 Figure S8.1: Forest plot for outcome = Morbidity: Hypoglycaemia

Study or Subgroup	0.1 U/kg/h IV Events	0.1 U/kg/h IV Total	1.0-2.2 U/kg SC every 3 hours Events	1.0-2.2 U/kg SC every 3 hours Total	Weight	Risk Ratio M-H, Random, 95% CI
Edwards 1977	3	10	4	10	100.0%	0.75 [0.22, 2.52]
**Total (95% CI)**		**10**		**10**	**100.0%**	**0.75 [0.22, 2.52]**
Total events	3		4			

Heterogeneity: Not applicable
Test for overall effect: Z = 0.46 (P = 0.64)

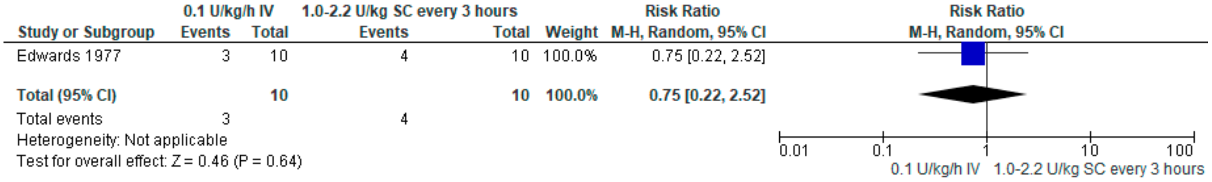

Supplementary S4 Figure S8.2: Forest plot for outcome = Morbidity: Hypokalaemia

Study or Subgroup	0.1 U/kg/h IV Events	0.1 U/kg/h IV Total	1.0-2.2 U/kg SC every 3 hours Events	1.0-2.2 U/kg SC every 3 hours Total	Weight	Risk Ratio M-H, Random, 95% CI
Edwards 1977	3	30	5	30	100.0%	0.60 [0.16, 2.29]
**Total (95% CI)**		**30**		**30**	**100.0%**	**0.60 [0.16, 2.29]**
Total events	3		5			

Heterogeneity: Not applicable
Test for overall effect: Z = 0.75 (P = 0.45)

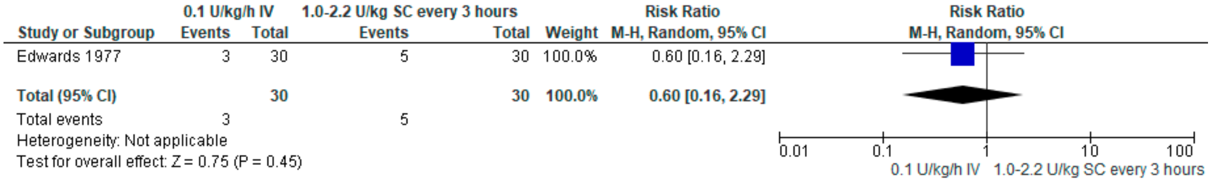

Supplementary S4 Figure S9: Dose: 0.25 U/kg IV bolus + 0.1 U/kg/h IV vs. 2.0 U/kg IV + SC

Study or Subgroup	0.25 U/kg IV bolus + 0.1 U/kg/h IV Insulin Events	0.25 U/kg IV bolus + 0.1 U/kg/h IV Insulin Total	2.0 U/kg IV + SC Insulin Events	2.0 U/kg IV + SC Insulin Total	Weight	Risk Ratio M-H, Random, 95% CI
Perkin 1979	0	25	3	33	100.0%	0.19 [0.01, 3.46]
**Total (95% CI)**		**25**		**33**	**100.0%**	**0.19 [0.01, 3.46]**
Total events	0		3			

Heterogeneity: Not applicable
Test for overall effect: Z = 1.13 (P = 0.26)

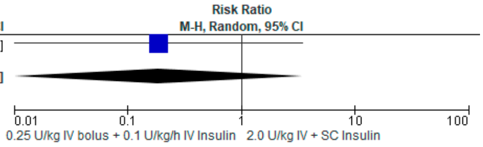

Supplementary S4 Figure S9.1: Forest plot for outcome = Morbidity: Hypoglycaemia

Study or Subgroup	0.25 U/kg IV bolus + 0.1 U/kg/h IV Insulin Events	0.25 U/kg IV bolus + 0.1 U/kg/h IV Insulin Total	2.0 U/kg IV + SC Insulin Events	2.0 U/kg IV + SC Insulin Total	Weight	Risk Ratio M-H, Random, 95% CI
Perkin 1979	0	25	2	33	100.0%	0.26 [0.01, 5.22]
**Total (95% CI)**		**25**		**33**	**100.0%**	**0.26 [0.01, 5.22]**
Total events	0		2			

Heterogeneity: Not applicable
Test for overall effect: Z = 0.88 (P = 0.38)

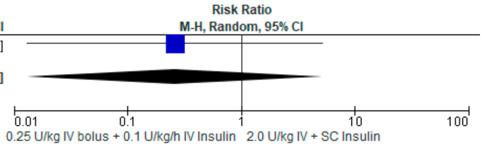

Supplementary S4 Figure S9.2: Forest plot for outcome = Morbidity: Hypokalaemia

Study or Subgroup	0.25 U/kg IV bolus + 0.1 U/kg/h IV Insulin Events	0.25 U/kg IV bolus + 0.1 U/kg/h IV Insulin Total	2.0 U/kg IV + SC Insulin Events	2.0 U/kg IV + SC Insulin Total	Weight	Risk Ratio M-H, Random, 95% CI
Perkin 1979	0	50	5	66	100.0%	0.12 [0.01, 2.11]
**Total (95% CI)**		**50**		**66**	**100.0%**	**0.12 [0.01, 2.11]**
Total events	0		5			

Heterogeneity: Not applicable
Test for overall effect: Z = 1.45 (P = 0.15)

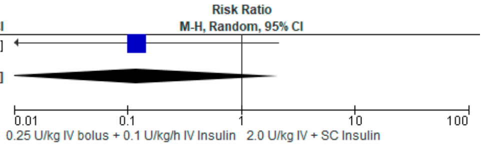

Supplementary S5: Outcomes for GRADE

Supplementary S5 Table S1: Dose: 0.05 U/kg/h IV insulin vs 0.1 U/kg/h IV insulin

Certainty assessment							№ of patients		Effect		Certainty	Importance
№ of studies	Study design	Risk of bias	Inconsistency	Indirectness	Imprecision	Other considerations	Dose: 0.05 U/kg/h IV	0.1 U/kg/h IV	Relative (95% CI)	Absolute (95% CI)		
**Morbidity: cerebral injury (based on the criteria given by Muir, et al. 2004)**												
3	randomised trials	very serious[a]	not serious	not serious	very serious[b]	none	0/70 (0.0%)	1/70 (1.4%)	RR 0.33 (0.01 to 7.81)	10 fewer per 1,000 (from 14 fewer to 97 more)	⊕◯◯◯ Very low[a,b]	CRITICAL
**Morbidity: Hypoglycemia (blood glucose level <3.3-3.9 mmol/L)**												
3	randomised trials	serious[c]	not serious	not serious	serious[d]	none	6/70 (8.6%)	17/70 (24.3%)	RR 0.39 (0.18 to 0.88)	148 fewer per 1,000 (from 199 fewer to 29 fewer)	⊕⊕◯◯ Low[c,d]	CRITICAL
**Morbidity: Hypokalemia (serum potassium <3.5 mmol/L and/or suggestive electrocardiographic changes)**												

**3**	randomised trials	not serious[e]	not serious	not serious	serious[d]	none	17/70 (24.3%)	32/70 (45.7%)	RR 0.54 (0.33 to 0.89)	210 fewer per 1,000 (from 306 fewer to 50 fewer)	⊕⊕⊕◯ Moderate[d,e]	CRITICAL
**Overall Morbidity (composite: cerebral injury, hypoglycemia and hypokalemia)**												
**3**	randomised trials	very serious[a,c,e]	not serious	not serious	serious[d]	none	23/210 (11.0%)	50/210 (23.8%)	RR 0.47 (0.30 to 0.74)	126 fewer per 1,000 (from 167 fewer to 62 fewer)	⊕◯◯◯ Very low[a,c,d,e]	CRITICAL

CI: confidence interval; **RR:** risk ratio

Explanations

a. The study with the highest weight had a high risk of bias in domain 4 and 'some concerns' in domain 5.
b. Wide CIs crossing the line of null effect and sample size not meeting the optimal information size.
c. One study had some concerns in three domains (70% weightage), two studies showed low risk of bias.
d. Small sample size not meeting optimal information size.
e. One study had some concerns in domain 2 and 5 (18% weightage), the other two showed low risk of bias.

Supplementary S5 Table S2: Dose: 0.05-0.1 U/kg/h IV Insulin vs >0.1 U/kg/2h SC Insulin

Certainty assessment	№ of patients	Effect	Certainty	

**№ of studies**	Study design	Risk of bias	Inconsistency	Indirectness	Imprecision	Other considerations	Dose: 0.05-0.1 U/kg/h IV	>0.1 U/kg SC	Relative (95% CI)	Absolute (95% CI)		**Importance**
**Morbidity: Hypoglycemia (glucose level of <3.3 mmol/L)**												
**1**	randomised trials	serious[a]	not serious[b]	not serious[c]	very serious[d]	none	6/30 (20.0%)	4/30 (13.3%)	RR 1.50 (0.47 to 4.78)	67 more per 1,000 (from 71 fewer to 504 more)	⊕◯◯◯ Very low[a,b,c,d]	CRITICAL
**Hospital Stay (number of days of hospitalization)**												
**1**	randomised trials	serious[a]	very serious[e]	not serious[c]	very serious[d]	none	50	50	-	MD 10.44 higher (17.79 lower to 38.66 higher)	⊕◯◯◯ Very low[a,c,d,e]	CRITICAL

CI: confidence interval; **MD:** mean difference; **RR:** risk ratio

Explanations

a. Study included shows 'some concerns' on risk of bias assessment on domains 1, 2 and 5.
b. Cannot comment on inconsistency as this is a single study. Statistical test for heterogeneity not given.
c. Study addresses the PICO.
d. Wide CI including null effect and sample size not meeting optimal information size.
e. I2 >90%, p-value significant, CIs do not overlap and point estimates vary widely.

N.B. Intervention and control arms switched for meta-analysis

Supplementary S5 Table S3: Route: 0.05-0.1 U/kg/h IV insulin vs >0.1 U/kg SC insulin every 2 hours

Certainty assessment							№ of patients		Effect		Certainty	Importance
**№ of studies**	Study design	Risk of bias	Inconsistency	Indirectness	Imprecision	Other considerations	Dose: 0.05-0.1 U/kg/h IV	>0.1 U/kg SC	Relative (95% CI)	Absolute (95% CI)		
**Morbidity: Hypoglycemia (glucose level of <3.3 mmol/L)**												
**1**	randomised trials	serious[a]	not serious[b]	not serious[c]	very serious[d]	none	6/30 (20.0%)	4/30 (13.3%)	RR 1.50 (0.47 to 4.78)	67 more per 1,000 (from 71 fewer to 504 more)	⨁◯◯◯ Very low[a,b,c,d]	CRITICAL
**Hospital Stay (number of days of hospitalization)**												
**1**	randomised trials	serious[a]	very serious[e]	not serious[c]	very serious[d]	none	50	50	-	MD 10.44 higher (17.79 lower to 38.66 higher)	⨁◯◯◯ Very low[a,c,d,e]	CRITICAL

CI: confidence interval; **MD:** mean difference; **RR:** risk ratio

Explanations

a. Study included shows 'some concerns' on risk of bias assessment on domains 1, 2 and 5.
b. Cannot comment on inconsistency as this is a single study. Statistical test for heterogeneity not given.
c. Study addresses the PICO.
d. Wide CI including null effect and sample size not meeting optimal information size.
e. I2 >90%, p-value significant, CIs do not overlap and point estimates vary widely.

N.B. Intervention and control arms switched for meta-analysis

Supplementary S5 Table S4: Frequency of Insulin Delivery: Every 2hr vs Continuous Infusion

Certainty assessment							№ of patients		Effect		Certainty	Importance
**№ of studies**	Study design	Risk of bias	Inconsistency	Indirectness	Imprecision	Other considerations	Frequency of Insulin Delivery: Every 2 hours SC	Continuous IV Infusion	Relative (95% CI)	Absolute (95% CI)		
**Morbidity: Hypoglycemia (glucose level of <3.3 mmol/L)**												
**1**	randomised trials	serious[a]	not serious[b]	not serious	very serious[c]	none	4/30 (13.3%)	6/30 (20.0%)	RR 0.67 (0.21 to 2.13)	66 fewer per 1,000 (from 158 fewer to 226 more)	⨁◯◯◯ Very low[a,b,c]	CRITICAL
**Hospital Stay (number of days of hospitalization)**												
**1**	randomised trials	serious[d]	very serious[e]	not serious	very serious[c]	none	50	50	-	MD 10.44 lower (38.66 lower to 17.79 higher)	⨁◯◯◯ Very low[c,d,e]	CRITICAL

CI: confidence interval; **MD:** mean difference; **RR:** risk ratio

Explanations

a. Study included shows' some concerns' on risk of bias assessment domains 1, 2 and 5.
b. Cannot comment on inconsistency as this is a single study. Statistical test for heterogeneity not given.
c. Wide CI including null effect and sample size not meeting optimal information size.
d. Study included shows 'some concerns' on risk of bias assessment for all domains except domain 3.
e. I2 >90%, p-value significant, CIs do not overlap and point estimates vary widely.

Supplementary S5 Table S5: Dose: 0.05-0.1 U/kg/h IV + 0.5U/kg SC vs. 0.05-0.1 U/kg/h IV

**Certainty assessment**							**№ of patients**		**Effect**		**Certainty**	**Importance**
**№ of studies**	Study design	Risk of bias	Inconsistency	Indirectness	Imprecision	Other considerations	Dose: 0.05-0.1 U/kg/h IV + 0.5U/kg SC	0.05-0.1 U/kg/h IV	Relative (95% CI)	Absolute (95% CI)		
**Morbidity: cerebral injury (based on bedside examination and assessment of the neurological condition)**												
**1**	randomised trials	serious[a]	not serious[b]	not serious	very serious[c]	none	15/72 (20.8%)	9/36 (25.0%)	RR 0.83 (0.40 to 1.72)	43 fewer per 1,000 (from 150 fewer to 180 more)	⊕◯◯◯ Very low[a,b,c]	CRITICAL
**Morbidity: Hypoglycemia (glucose <3.89 mmol/L)**												
**1**	randomised trials	serious[a]	not serious[b]	not serious	very serious[c]	none	11/72 (15.3%)	3/36 (8.3%)	RR 1.83 (0.55 to 6.16)	69 more per 1,000 (from 37 fewer to 430 more)	⊕◯◯◯ Very low[a,b,c]	CRITICAL
**Morbidity: Hypokalemia (potassium <3.5 mmol/L)**												
**1**	randomised trials	serious[d]	not serious[b]	not serious	very serious[c]	none	15/72 (20.8%)	7/36 (19.4%)	RR 1.07 (0.48	14 more per 1,000	⊕◯◯◯	CRITICAL

									to 2.39)	(from 101 fewer to 270 more)	Very low[b,c,d]	
**Overall Morbidity (composite: cerebral injury, hypoglycemia and hypokalemia)**												
**1**	randomised trials	serious[a,d]	not serious[b]	not serious	very serious[c]	none	41/216 (19.0%)	19/108 (17.6%)	RR 1.08 (0.66 to 1.77)	14 more per 1,000 (from 60 fewer to 135 more)	⊕◯◯◯ Very low[a,b,c,d]	CRITICAL

CI: confidence interval; **RR:** risk ratio

Explanations

a. The study included shows 'some concerns' in ROB under the following domains: Randomisation process, Deviations from the intended interventions, Measurement of the outcome, and Selection of the reported result

b. Cannot comment on inconsistency due to the presence of only one study for this outcome.

c. Wide CI including null effect and sample size not meeting optimal information size.

d. The study included shows 'some concerns' in ROB under the following domains: Randomisation process, Deviations from the intended interventions, and Selection of the reported result

Supplementary S5 Table S6: Dose: 0.1 U/kg/2h IM vs. 1.0 U/kg/4h SC + IV

Certainty assessment							№ of patients		Effect		Certainty	Importance
№ of studies	Study design	Risk of bias	Inconsistency	Indirectness	Imprecision	Other considerations	0.1 U/kg/2h IM	1.0 U/kg/4h SC + IV	Relative (95% CI)	Absolute (95% CI)		
**Morbidity: cerebral injury (not defined)**												
**1**	randomised trials	serious[a]	not serious	not serious	serious[b]	none	0/7 (0.0%)	0/6 (0.0%)	not estimable		⊕⊕◯◯ Low[a,b]	CRITICAL
**Morbidity: Hypoglycemia (not defined)**												
**1**	randomised trials	serious[c]	not serious	not serious	serious[b]	none	0/7 (0.0%)	0/6 (0.0%)	not estimable		⊕⊕◯◯ Low[b,c]	CRITICAL
**Morbidity: Hypokalemia (not defined)**												
**1**	randomised trials	serious[c]	not serious	not serious	serious[b]	none	0/7 (0.0%)	0/6 (0.0%)	not estimable		⊕⊕◯◯ Low[b,c]	CRITICAL
**Overall Morbidity (composite of cerebral injury, hypoglycemia, hypokalemia)**												
**1**	randomised trials	serious[a,c]	not serious	not serious	serious[b]	none	0/21 (0.0%)	0/18 (0.0%)	not estimable		⊕⊕◯◯ Low[a,b,c]	CRITICAL
**Adverse Events (hypernatremia)**												
**1**	randomised trials	serious[c]	not serious	not serious	very serious[d]	none	1/7 (14.3%)	2/6 (33.3%)	RR 0.43 (0.05 to 3.64)	190 fewer per 1,000 (from 317 fewer to	⊕◯◯◯ Very low[c,d]	CRITICAL

										880 more)		

CI: confidence interval; **RR:** risk ratio

Explanations

a. Some concerns of bias in all domains except domain 3.
b. Small sample size not meeting optimal information size.
c. Some concerns of bias in domains 1, 2 and 5.
d. Wide CI crossing the line of null effect. Small sample size not meeting optimal information size.

Supplementary S5 Table S7: Dose: 0.1 U/kg/h IV vs. 1.0 U/kg/h IV

**Certainty assessment**							**№ of patients**		**Effect**		**Certainty**	**Importance**
**№ of studies**	Study design	Risk of bias	Inconsistency	Indirectness	Imprecision	Other considerations	Dose: 0.1 U/kg/h IV	1.0 U/kg/h IV	Relative (95% CI)	Absolute (95% CI)		
**Morbidity: Hypoglycemia (plasma glucose <5.6 mmol/L)**												
**1**	randomised trials	serious[a]	not serious	not serious	serious[b]	none	2/16 (12.5%)	12/16 (75.0%)	RR 0.17 (0.04 to 0.63)	622 fewer per 1,000 (from 720 fewer to 277 fewer)	⨁⨁◯◯ Low[a,b]	CRITICAL
**Morbidity: Hypokalemia (plasma potassium levels <3.4 mmol/L)**												
**1**	randomised trials	serious[a]	not serious	not serious	serious[b]	none	3/16 (18.8%)	10/16 (62.5%)	RR 0.30 (0.10 to 0.89)	438 fewer per 1,000 (from 563 fewer to 69 fewer)	⨁⨁◯◯ Low[a,b]	CRITICAL
**Overall Morbidity (composite: hypoglycemia and hypokalemia)**												
**1**	randomised trials	serious[a]	not serious	not serious	serious[b]	none	5/32 (15.6%)	22/32 (68.8%)	RR 0.23 (0.10 to 0.53)	529 fewer per 1,000	⨁⨁◯◯ Low[a,b]	CRITICAL

										(from 619 fewer to 323 fewer)		
**Mortality**												
**1**	randomised trials	serious[a]	not serious	not serious	serious[b]	none	0/16 (0.0%)	0/16 (0.0%)	not estimable		⊕⊕◯◯ Low[a,b]	CRITICAL

CI: confidence interval; **RR:** risk ratio

Explanations

a. The study has some concerns of bias in domains 1, 2 and 5.

b. Sample size not meeting the optimal information size.

Supplementary S5 Table S8: Dose: 0.1 U/kg/h IV vs. 1.0-2.2 U/kg SC every 3 hours

Certainty assessment							№ of patients		Effect		Certainty	Importance
**№ of studies**	Study design	Risk of bias	Inconsistency	Indirectness	Imprecision	Other considerations	Dose: 0.1 U/kg/h IV	1.0-2.2 U/kg SC every 3 hours	Relative (95% CI)	Absolute (95% CI)		
**Morbidity: Cerebral injury (not defined)**												
**1**	randomised trials	very serious[a]	not serious	not serious	serious[b]	none	0/10 (0.0%)	0/10 (0.0%)	not estimable		⨁◯◯◯ Very low[a,b]	CRITICAL
**Morbidity: Hypoglycemia (Plasma glucose: 48 mg/dL)**												
**1**	randomised trials	serious[c]	not serious	not serious	very serious[d]	none	0/10 (0.0%)	1/10 (10.0%)	RR 0.33 (0.02 to 7.32)	67 fewer per 1,000 (from 98 fewer to 632 more)	⨁◯◯◯ Very low[c,d]	CRITICAL
**Morbidity: Hypokalemia (Serum potassium <3.5 mEq/L)**												
**1**	randomised trials	serious[e]	not serious	not serious	very serious[d]	none	3/10 (30.0%)	4/10 (40.0%)	RR 0.75 (0.22 to 2.52)	100 fewer per 1,000 (from 312 fewer to 608 more)	⨁◯◯◯ Very low[d,e]	CRITICAL

**Overall Morbidity (composite: cerebral injury, hypoglycemia, hypokalemia)**												
**1**	randomised trials	very serious[a,c,e]	not serious	not serious	very serious[d]	none	3/30 (10.0%)	5/30 (16.7%)	RR 0.60 (0.16 to 2.29)	67 fewer per 1,000 (from 140 fewer to 215 more)	⨁◯◯◯ Very low[a,c,d,e]	CRITICAL

CI: confidence interval; **RR:** risk ratio

Explanations

a. Study shows high risk of bias in domain 4.
b. Small sample size not meeting optimal information size.
c. Study shows some concerns of bias in domains 1, 4 and 5.
d. Wide CI crossing the line of null effect. Small sample size not meeting optimal information size.
e. Study shows some concerns of bias in domains 1 and 5.

Supplementary S5 Table S9: Dose: 0.25 U/kg IV bolus + 0.1 U/kg/h IV vs. 2.0 U/kg IV + SC

Certainty assessment							№ of patients		Effect		Certainty	Importance
**№ of studies**	Study design	Risk of bias	Inconsistency	Indirectness	Imprecision	Other considerations	Dose: 0.25 U/kg IV bolus + 0.1 U/kg/h IV	2.0 U/kg IV + SC	Relative (95% CI)	Absolute (95% CI)		
**Morbidity: Hypoglycemia (Serum glucose <2.2 mmol/L)**												
**1**	randomised trials	serious[a]	not serious[b]	not serious	very serious[c]	none	0/25 (0.0%)	3/33 (9.1 %)	RR 0.19 (0.01 to 3.46)	74 fewer per 1,000 (from 90 fewer to 224 more)	⊕◯◯◯ Very low[a,b,c]	CRITICAL
**Morbidity: Hypokalemia (Potassium <3 mmol/L)**												
**1**	randomised trials	very serious[d]	not serious	not serious	very serious[c]	none	0/25 (0.0%)	2/33 (6.1 %)	RR 0.26 (0.01 to 5.22)	45 fewer per 1,000 (from 60 fewer to 256 more)	⊕◯◯◯ Very low[c,d]	CRITICAL
**Overall Morbidity (composite: hypoglycemia and hypokalemia)**												
**1**	randomised trials	very serious[a,d]	not serious	not serious	very serious[c]	none	0/50 (0.0%)	5/66 (7.6 %)	RR 0.12 (0.01 to 2.11)	67 fewer per 1,000 (from 75 fewer to	⊕◯◯◯ Very low[a,c,d]	CRITICAL

										84 more)		

CI: confidence interval; **RR:** risk ratio

Explanations

a. Some concerns of bias in domains 1 and 5.

b. Cannot comment on inconsistency as this is a single study. Statistical test for heterogeneity not given.

c. Wide CI crossing the line of null effect. Small sample size not meeting optimal information size.

d. High risk of bias in domain 1 and some concerns in domain 5.

Supplementary S5 Table S10: Dose: 0.1 U/kg/h IV vs. 0.9-1.8 U/kg/h IV + SC

Certainty assessment							№ of patients		Effect		Certainty	Importance
**№ of studies**	Study design	Risk of bias	Inconsistency	Indirectness	Imprecision	Other considerations	Dose: 0.1 U/kg/h IV	0.9-1.8 U/kg/h IV + SC	Relative (95% CI)	Absolute (95% CI)		
**Morbidity: Hypoglycemia (not defined)**												
**1**	randomised trials	very serious[a]	not serious[b]	not serious	serious[c]	none	0/9 (0.0%)	0/9 (0.0%)	not estimable		⨁◯◯◯ Very low[a,b,c]	CRITICAL
**Morbidity: Hypokalemia (serum potassium level <3.5 mmol/L or ECG 'T wave' changes)**												
**1**	randomised trials	very serious[a]	not serious[b]	not serious	serious[c]	none	0/9 (0.0%)	0/9 (0.0%)	not estimable		⨁◯◯◯ Very low[a,b,c]	CRITICAL
**Overall Morbidity (composite: hypoglycemia and hypokalemia)**												
**1**	randomised trials	very serious[a]	not serious[b]	not serious	serious[c]	none	0/18 (0.0%)	0/18 (0.0%)	not estimable		⨁◯◯◯ Very low[a,b,c]	CRITICAL

CI: confidence interval; **RR:** risk ratio

Explanations

a. The study included shows overall 'high risk' in ROB, showing 'high risk' under the Randomisation process domain and 'some concerns' under Deviations from the intended interventions, and Selection of the reported result domains.
b. Cannot comment on inconsistency due to the presence of only one study for this outcome.
c. Small sample size not meeting optimal information size.

Supplementary S5 Table S11: 0.1 U/kg/h IV with bolus dose vs. 0.1 U/kg/h IV without bolus dose

Certainty assessment							№ of patients		Effect		Certainty	Importance
**№ of studies**	Study design	Risk of bias	Inconsistency	Indirectness	Imprecision	Other considerations	0.1 U/kg/h IV with bolus dose	0.1 U/kg/h IV with no bolus dose	Relative (95% CI)	Absolute (95% CI)		
**Morbidity: cerebral injury**												
**1**	randomised trials	very serious[a]	not serious[b]	not serious	serious[c]	none	0/24 (0.0%)	0/32 (0.0%)	not estimable		⨁◯◯◯ Very low[a,b,c]	CRITICAL

CI: confidence interval; **RR:** risk ratio

Explanations

a. The study included shows overall 'high risk' in ROB, showing 'high risk' under the Randomisation process domain and 'some concerns' under Deviations from the intended interventions, Measurement of the outcome, and Selection of the reported result domains.

b. Cannot comment on inconsistency due to the presence of only one study for this outcome.

c. Small sample size not meeting optimal information size.
